# Non‐Hydrolytic β‐Lactam Antibiotic Fragmentation by l,d‐Transpeptidases and Serine β‐Lactamase Cysteine Variants

**DOI:** 10.1002/anie.201809424

**Published:** 2019-01-21

**Authors:** Christopher T. Lohans, H. T. Henry Chan, Tika R. Malla, Kiran Kumar, Jos J. A. G. Kamps, Darius J. B. McArdle, Emma van Groesen, Mariska de Munnik, Catherine L. Tooke, James Spencer, Robert S. Paton, Jürgen Brem, Christopher J. Schofield

**Affiliations:** ^1^ Department of Chemistry University of Oxford Oxford OX1 3TA UK; ^2^ School of Cellular and Molecular Medicine University of Bristol Bristol BS8 1TD UK

**Keywords:** antibiotic resistance, fragmentation, hydrolases, transpeptidases, β-lactamases

## Abstract

Enzymes often use nucleophilic serine, threonine, and cysteine residues to achieve the same type of reaction; the underlying reasons for this are not understood. While bacterial d,d‐transpeptidases (penicillin‐binding proteins) employ a nucleophilic serine, l,d‐transpeptidases use a nucleophilic cysteine. The covalent complexes formed by l,d‐transpeptidases with some β‐lactam antibiotics undergo non‐hydrolytic fragmentation. This is not usually observed for penicillin‐binding proteins, or for the related serine β‐lactamases. Replacement of the nucleophilic serine of serine β‐lactamases with cysteine yields enzymes which fragment β‐lactams via a similar mechanism as the l,d‐transpeptidases, implying the different reaction outcomes are principally due to the formation of thioester versus ester intermediates. The results highlight fundamental differences in the reactivity of nucleophilic serine and cysteine enzymes, and imply new possibilities for the inhibition of nucleophilic enzymes.

Nature employs enzymes with nucleophilic serine, threonine, or cysteine residues to catalyze closely related reactions. Well‐known examples of this are the serine and cysteine proteases, in which these different nucleophilic residues are used to cleave peptide bonds. An analogous situation exists for the transpeptidase enzymes involved in bacterial peptidoglycan biosynthesis; the d,d‐transpeptidases (or penicillin‐binding proteins; PBPs) employ a nucleophilic serine, whereas the l,d‐transpeptidases (Ldts) employ a nucleophilic cysteine. The reasons for the use of a particular nucleophilic residue in a particular enzyme context are not understood.

Bacterial peptidoglycan biosynthesis is an antibiotic target of immense clinical importance.^1^, ^2^, ^3^ Peptidoglycan, an extracellular network of polysaccharides and cross‐linked peptides, plays a critical structural role in the bacterial cell wall.^4^ In many Gram‐negative bacteria, cross‐links occur primarily between *meso*‐diaminopimelate (*meso*‐Dap) and d‐Ala residues. Formation of these *meso*‐Dap‐d‐Ala cross‐links is catalyzed by the d,d‐transpeptidase activities of the PBPs. However, the peptidoglycan of some bacteria (for example, *Mycobacterium tuberculosis* in stationary phase) consists substantially of cross‐links formed between *meso*‐Dap residues, catalyzed by the Ldts.^5^, ^6^ The transpeptidase activities of PBPs and Ldts both proceed via acyl‐enzyme substrate complexes formed by reaction with a donor substrate peptide; the acyl‐enzyme complex subsequently reacts with an amino group of an acceptor peptide to give the cross‐linked product. While the acyl‐enzyme complex is formed by reaction with a nucleophilic serine residue in the PBPs, the Ldts instead employ a nucleophilic cysteine.

The PBPs are established targets of β‐lactam drugs, the most widely used class of antibiotic.^7^ β‐Lactams (Figure 1 A) inhibit PBPs by covalently modifying the nucleophilic serine (Figure 1 B). The use of β‐lactams is compromised by bacterial resistance mechanisms, most importantly the production of serine β‐lactamases (SBLs). The SBL mechanism involves the reaction of the β‐lactam ring with a nucleophilic serine to form an acyl‐enzyme complex (Figure 1 B), which is then efficiently hydrolyzed. Three of the five clinically used SBL inhibitors (for example, clavulanic acid) react irreversibly to form hydrolytically stable acyl‐enzyme complexes, whilst the other two (avibactam and vaborbactam) react reversibly.^8^

**Figure 1:**
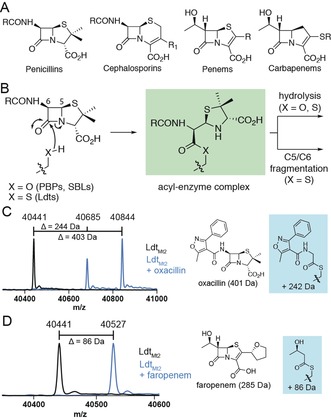
A) The penicillin, cephalosporin, penem, and carbapenem β‐lactam classes. B) Reaction of the nucleophilic serine (PBPs, SBLs) or cysteine (Ldts) with a generic penicillin, forming an acyl‐enzyme complex which can undergo hydrolysis or C5–C6 fragmentation. MS data for Ldt_Mt2_ (4 μm) incubated with a 100‐fold excess of C) oxacillin for 2 h, and D) faropenem for 10 min. Black spectra: Ldt_Mt2_; blue spectra: Ldt_Mt2_ with the indicated β‐lactam.

The nucleophilic cysteine of the Ldts can also be acylated by β‐lactams (Figure 1 B).^9^, ^10^ In particular, carbapenems and penems (Figure 1 A) are potent Ldt inhibitors,^9^, ^11^ and manifest antibiotic activity against *M. tuberculosis*.^12^, ^13^ However, the Ldts are often considered insensitive to the other classes of β‐lactam antibiotics (for example, penicillins).^6^, ^14^, ^15^

Recent work has suggested that the acyl‐enzyme complexes formed by some β‐lactams with Ldts behave differently than the analogous complexes formed with PBPs or SBLs (Figure 1 B).^9^, ^12^, ^16^ However, it is unclear whether the difference in reactivity arises from the cysteine‐for‐serine substitution, or if it is due to other active site variations. Based on studies of Ldt_Mt2_ (from *M. tuberculosis*) and cysteine variants of the OXA‐48 and KPC‐2 SBLs, we report that the difference in reactivity is principally due to the identity of the nucleophilic residue, and arises from the different chemical properties of the ester and thioester intermediates. This is a rare example where the identity of the nucleophilic residue changes the reaction outcome of an enzyme. The results suggest that the biological use of specific nucleophiles may be related to function, and raise the potential of developing new nucleophilic‐enzyme inhibitors (including for serine and cysteine proteases) and antibiotics, i.e., which are poised to undergo fragmentation reactions, yielding stable acyl‐enzyme complexes.

To compare the reactivity of the Ldts with the nucleophilic serine enzymes (i.e., PBPs and SBLs), mass spectrometry (MS) was used to monitor the adducts formed by Ldt_Mt2_ with a panel of β‐lactam antibiotics. While screening representative penicillins, mass shifts were observed that are consistent with the formation of acyl‐enzyme complexes with oxacillin, piperacillin, and ticarcillin, but not with ampicillin (Figure 1 C and Supporting Information, Figure S1). Over time, a new species 159 Da smaller than the intact acyl‐enzyme complexes accumulated for Ldt_Mt2_ in the presence of ampicillin, oxacillin, and piperacillin (Figure 1 C, and Supporting Information, Figures S1 and S2). This mass difference is consistent with the cleavage of the C5–C6 bond of the penicillin‐derived Ldt_Mt2_ acyl‐enzyme complex, yielding Ldt_Mt2_ modified by an acetyl group bearing the penicillin C6 side chain. This is similar to what has been observed previously for ampicillin with other Ldts.^16^, ^17^ In the case of faropenem, a peak corresponding to Ldt_Mt2_ acylated with a 3‐hydroxybutyrate fragment was observed (Figure 1 D).^9^, ^12^ A similar adduct was observed following treatment of Ldt_Mt2_ with the penem sulopenem (Supporting Information, Figure S1). Incubation of Ldt_Mt2_ with the cephalosporins cephalothin, cefotaxime, and cefapirin resulted in adducts in which the C3′ leaving groups were lost (Supporting Information, Figure S1). Fragmentation was not observed for the carbapenems meropenem, imipenem, and ertapenem (Supporting Information, Figure S1), and acylation was not observed for the monobactam aztreonam and the oxacephem moxalactam. NMR experiments indicated that cephalothin, meropenem, aztreonam, and moxalactam were not efficiently degraded by Ldt_Mt2_ under the MS assay conditions. No acylation of the Ldt_Mt2_ C354S variant (where the nucleophilic cysteine is replaced with a serine) by β‐lactams was observed under the conditions used for MS (Supporting Information, Figure S3).

The fragmentation of penicillins and penems with Ldt_Mt2_, which is not typically observed for PBPs and SBLs, may result from the different chemical properties of the thioester (Ldts) and ester (PBPs and SBLs) acyl‐enzyme complexes. Alternatively, these differences could arise from other features of the active site; for example, there is less than 25 % sequence identity between PBP5 from *Pseudomonas aeruginosa* and Ldt_Mt2_ from *M. tuberculosis* (active sites are compared in Figure S4 in the Supporting Information). To investigate, variants of the clinically important OXA‐48 and KPC‐2 SBLs were prepared in which the nucleophilic serine was replaced with a cysteine. Consistent with previous reports on the cysteine variants of SBLs,^18^, ^19^, ^20^ OXA‐48 S70C (*k*_cat_/*K*_M_ 0.098±0.017 μm ^−1^ s^−1^ with the fluorogenic cephem substrate FC5)^21^ was not as catalytically active as the wild‐type enzyme (*k*_cat_/*K*_M_ 0.36±0.06 μm ^−1^ s^−1^ with FC5; Supporting Information, Table S1). However, the impact of this substitution on the enzymatic mechanism of the SBLs has not been previously described.

Following incubation of the penicillin ampicillin with wild‐type OXA‐48 or KPC‐2, only hydrolysis was observed by NMR (Figure 2 A). While OXA‐48 S70C and KPC‐2 S70C also catalyzed ampicillin hydrolysis, additional products were formed (Figure 2 A). Based on further NMR and MS analyses, these products were assigned as the d‐phenylglycylglycine dipeptide (derived in part from C6 and C7 of ampicillin) and a dihydrothiazole (derived from the ampicillin thiazolidine ring) (Figure 2 B, Supporting Information, Figures S5 and S6, and Tables S2–S5). The assignment of d‐phenylglycylglycine was validated by comparison with a synthetic standard (Supporting Information, Figure S7). Treatment of other penicillins (for example, piperacillin, oxacillin, and ticarcillin) with OXA‐48 S70C and KPC‐2 S70C resulted in the formation of both hydrolysis and C5–C6 fragmentation products (Figure 2 C and Supporting Information, Figure S8). While Ldt_Mt2_‐catalyzed penicillin fragmentation could not be observed by NMR (Supporting Information, Figure S8), MS analyses indicated that OXA‐48 S70C and Ldt_Mt2_ form the same C5–C6 fragmentation products (Figure 1 C and Supporting Information, Figure S9). Although the relative levels of hydrolysis and C5–C6 fragmentation were similar for both OXA‐48 S70C and KPC‐2 S70C, Ldt_Mt2_ was observed to favor hydrolysis of the penicillin β‐lactam (contrasting with Ldt_fm_ from *Enterococcus faecium*, for which fragmentation is reported to predominate).^16^

**Figure 2:**
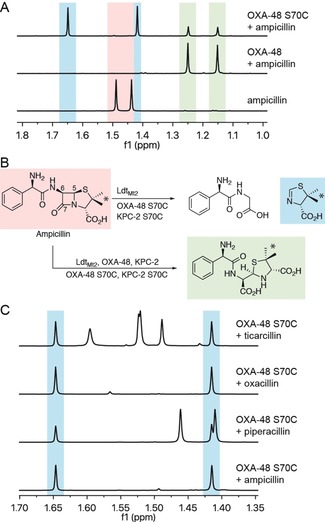
Reaction of penicillins with Ldt_Mt2_ and SBL thiol variants. A) ^1^H NMR spectra (700 MHz) of methyl group resonances for products formed upon addition of ampicillin to OXA‐48 S70C, compared to wild‐type OXA‐48. These signals correspond to methyl groups indicated by the asterisks in panel B. B) Scheme showing ampicillin‐derived fragmentation products formed by Ldt_Mt2_ and SBL thiol variants, and hydrolysis products formed by wild‐type SBLs, SBL thiol variants, and Ldt_Mt2_. C) ^1^H NMR spectra (600 MHz) showing the formation of the same dihydrothiazole fragmentation product upon incubation of OXA‐48 S70C with ampicillin, piperacillin, oxacillin, and ticarcillin (see also Figure S8 in the Supporting Information). Note the other signals present arise from intact penicillins and hydrolyzed products.

Treatment of faropenem with OXA‐48 S70C or KPC‐2 S70C yielded different products than the hydrolysis products formed by wild‐type OXA‐48 and KPC‐2 (Figure 3 A and Supporting Information, S10). Based on NMR and MS, these products were assigned as 3‐hydroxybutyrate (derived in part from C6 and C7 of faropenem) and a thiazole (derived from the dihydrothiazole ring of faropenem) (Figure 3 B, Supporting Information, Figure S10 and Tables S6–S9). Further NMR analyses demonstrated that the same faropenem‐derived thiazole is formed by Ldt_Mt2_; enzyme‐catalyzed faropenem hydrolysis was not observed for Ldt_Mt2_ or OXA‐48 S70C (Supporting Information, Figure S11).

**Figure 3:**
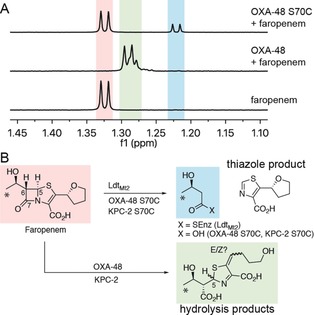
Reaction of faropenem with Ldt_Mt2_ and SBL thiol variants. A) ^1^H NMR spectra (600 MHz) showing the methyl group resonances of products formed upon addition of faropenem to OXA‐48 S70C and OXA‐48. Note, the two doublets (*δ* 1.28 ppm, 1.29 ppm) observed for the faropenem hydrolysis product were assigned as C5 epimers (see Table S7 in the Supporting Information), although double bond isomers are also possible. The highlighted signals correspond to methyl groups indicated by asterisks in panel B. B) Scheme showing the C5–C6 faropenem fragmentation products observed for Ldt_Mt2_ and SBL thiol variants, as compared to the hydrolysis products observed for wild‐type SBLs.

The products formed by Ldt_Mt2_ and the SBL thiol variants with penicillins and penems are consistent with C5–C6 bond cleavage, and inform on the mechanism of fragmentation (Figure 4 A,B). The penicillin‐derived dihydrothiazole and faropenem‐derived thiazole products imply that the nitrogen/sulfur lone pairs in the thiazolidine (for penicillins) or dihydrothiazole (for faropenem) rings promote C5–C6 bond cleavage. Both reaction pathways likely proceed via a thioester‐enolate intermediate involving the nucleophilic cysteine. Formation of this thioester‐enolate is expected to be more energetically favorable than the formation of the analogous ester‐enolate (involving a nucleophilic serine). As the stability of these enolates appeared to relate to the acidity at the α‐position of the corresponding ester/thioester, calculations were performed to compare the p*K*_a_ values for the ester and thioester species derived from β‐lactam fragmentation (Supporting Information, Figure S12 and Tables S10–S13). The calculated p*K*_a_ values for the thioesters derived from ampicillin and faropenem fragmentation (as seen with the nucleophilic cysteine enzymes) are 7.2 and 6.4 units lower, respectively, than those for the analogous ester species (which were not seen for the nucleophilic serine enzymes) (Supporting Information, Table S11). These calculated values are consistent with experimentally determined p*K*_a_ values for ethyl acetate (p*K*_a_=25.6)^22^ and ethyl thioacetate (p*K*_a_=20.4–21.5).^23^ Thus, the favorable formation of thioester‐enolates may rationalize why enzymes with nucleophilic cysteine residues (for example, Ldts and SBL thiol variants) catalyze β‐lactam C5–C6 fragmentation, while those with nucleophilic serine residues (for example, PBPs and SBLs) typically do not.

**Figure 4:**
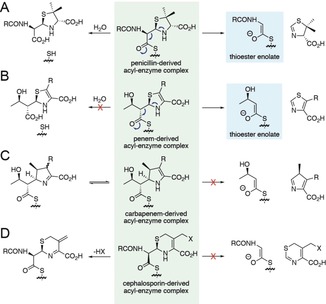
Comparison of the reactivities of the acyl‐enzyme complexes formed from Ldt_Mt2_ with different β‐lactam antibiotic sub‐classes. For the A) penicillins and B) penems, C5–C6 fragmentation gives rise to a thioester‐enolate. Note that penicillin hydrolysis occurs preferentially to fragmentation for Ldt_Mt2_ (Figure 2 and Supporting Information, Figure S8), while only fragmentation of faropenem was detected (Figure 3 and Supporting Information, Figure S11). C) C5–C6 fragmentation was not observed for the carbapenems (Supporting Information, Figure S1), potentially in part due to delocalization of the pyrroline nitrogen lone pair or due to tautomerization of the pyrroline ring (as well as the lack of a heteroatom at position 1 of the ring). Note the hypothetical product would be expected to tautomerize to a pyrrole. D) C6–C7 fragmentation was not observed for cephalosporins; however, elimination of the C3′ leaving group occurs (Supporting Information, Figure S1). Note that carbapenem and cephalosporin hydrolysis by Ldt_Mt2_ was not observed under our NMR conditions.

Fragmentation was not observed for the acyl‐enzyme complexes derived from carbapenems with Ldt_Mt2_ (Figure 4 C), contrasting with the rapid fragmentation of the penems faropenem and sulopenem, suggesting the heteroatom at position 1 is important. While the C5–C6 bond cleavage of the faropenem‐derived acyl‐enzyme complex forms an aromatic thiazole (Figure 4 B), the product immediately formed following C5–C6 cleavage of a carbapenem‐derived complex would not be aromatic (although it would likely rapidly tautomerize to give an aromatic pyrrole). Furthermore, the pyrroline ring of the carbapenem‐derived acyl‐enzyme complex can tautomerize, impacting on the availability of the β‐lactam‐derived nitrogen lone pair. Analogous C6–C7 bond cleavage of the cephalosporin‐derived acyl‐enzyme complexes was also not observed (Figure 4 D). Instead, elimination of the cephalosporin C3′ substituent occurs, limiting the ability of the nitrogen lone pair in the dihydrothiazine ring to promote C6–C7 cleavage.

These results imply that the nature of the nucleophilic residue can determine the fate of the acyl‐enzyme complex; however, other active site features can be relevant. As we and others have observed, the Ldt_Mt2_ C354S variant does not form covalent complexes with the carbapenems and penems tested.^12^ Furthermore, the C5–C6 fragmentation of benzylpenicillin has been described for a PBP from a *Streptomyces* spp.,^24^ while the formation of a thiazole product from faropenem was observed for some class C SBLs.^25^ It could be that as yet unidentified features in the active sites of some nucleophilic serine enzymes promote C5–C6 fragmentation. The acyl‐enzyme complexes of SBLs with some other β‐lactams favor alternative (i.e., not C5–C6) fragmentation reactions, as reported for the SBL inhibitors clavulanic acid, sulbactam, and tazobactam.^7^, ^8^ Alternative enzyme‐catalyzed mechanisms not involving C−C fragmentation have also been observed, including the degradation of carbapenems by class D SBLs via lactone formation.^26^

There are few examples where substitution of the nucleophilic residue determines the type of reaction catalyzed by an enzyme. While the wild‐type protease subtilisin catalyzes hydrolysis, the subtilisin S221C variant also manifests transpeptidase activity.^27^ More typically, substitution of a nucleophilic serine with cysteine can stabilize the acyl‐enzyme complex.^28^, ^29^ However, acylation by the natural substrate may not occur following the substitution of serine with cysteine (and vice versa), as observed by us (for Ldt_Mt2_ C354S) and others.^30^, ^31^

The Ldts are antibiotic targets in pathogens, notably *M. tuberculosis*.^6^, ^12^ These results indicate that the different reactivities of the Ldts with β‐lactam antibiotics, as compared to the PBPs and SBLs, are substantially governed by the nucleophilic cysteine, which facilitates fragmentation reactions not normally observed for enzymes employing nucleophilic serines. These fragmentation reactions can be rationalized mechanistically by the relatively favorable involvement of a thioester‐enolate intermediate in fragmentation. These results highlight a potential new approach for the development of antibiotics targeting the Ldts, for example, the identification of β‐lactams that undergo C5–C6 (or C6–C7 for cephalosporins) fragmentation to give stable acyl‐enzyme complexes. They may also inspire the development of related inhibitors targeting other classes of enzymes with nucleophilic cysteine residues (for example, the cysteine proteases). The potential for toxic and/or immunomodulatory effects of the β‐lactam fragments/complexes formed by the Ldts should also be considered.^32^

More generally, these results suggest that it is of interest to consider links between the biological roles of nucleophilic enzymes and the nature of the nucleophilic residue employed, including in the many biologically important serine/cysteine proteases and related enzymes (for example, those involved in ubiquitin biology).^33^ The enhanced nucleophilicity of thiols compared to alcohols, and the increased reactivity of thioesters versus esters are often cited as factors for the use of thiols in biological systems. Our results, combined with prior studies on the nucleophilic residues of PBPs and SBLs, imply that both the reaction rate (including if the reaction occurs or not), and the fate of the acyl‐enzyme complex depend on the identity of the nucleophile and the context in which the ester or thioester exists. Thus, the use of nucleophilic serine or cysteine residues may reflect chemical evolutionary pressures subject to their biological roles and the types of reactions catalyzed. The latter may include factors relating to competing pathways/by‐products, which the present work shows can differ with the different types of nucleophile.

## Conflict of interest

The authors declare no conflict of interest.

## Supporting information

As a service to our authors and readers, this journal provides supporting information supplied by the authors. Such materials are peer reviewed and may be re‐organized for online delivery, but are not copy‐edited or typeset. Technical support issues arising from supporting information (other than missing files) should be addressed to the authors.

Supplementary
