# Supplementary material for: Non‐Hydrolytic β‐Lactam Antibiotic Fragmentation by l,d‐Transpeptidases and Serine β‐Lactamase Cysteine Variants
Source: Angew Chem Int Ed Engl. 2019 Jan 21;58(7):1990–4. doi: 10.1002/anie.201809424 (PMC6391942; doi:10.1002/anie.201809424)
Supplement: Supplementary file 1 — Supplementary [file ANIE-58-1990-s001.pdf]

Angewandte Chemie
Eine Zeitschrift der Gesellschaft Deutscher Chemiker

Supporting Information

Non-Hydrolytic β-Lactam Antibiotic Fragmentation by L,D-Transpeptidases and Serine β-Lactamase Cysteine Variants

*Christopher T. Lohans[+], H. T. Henry Chan[+], Tika R. Malla, Kiran Kumar, Jos J. A. G. Kamps, Darius J. B. McArdle, Emma van Groesen, Mariska de Munnik, Catherine L. Tooke, James Spencer, Robert S. Paton, Jürgen Brem, and Christopher J. Schofield**

anie_201809424_sm_miscellaneous_information.pdf

Supporting Information Contents

Experimental S2
Figure S1. MS for the complexes derived from Ldt_{Mt2} with a panel of β-lactams S8
Figure S2. C5-C6 fragmentation of the oxacillin-Ldt_{Mt2} complex S10
Figure S3. Characterization of Ldt_{Mt2} C354S S11
Figure S4. Comparison of the active sites of Ldt_{Mt2} and PBP5 S12
Table S1. Michaelis-Menten kinetic constants for OXA-48 and OXA-48 S70C S13
Table S2. Chemical shift assignments for ampicillin S14
Table S3. Chemical shift assignments for the ampicillin-derived hydrolysis product S14
Table S4. Chemical shift assignments for the ampicillin-derived dipeptide S15
Table S5. Chemical shift assignments for the ampicillin-derived dihydrothiazole S15
Figure S5. Degradation of ampicillin by KPC-2 and KPC-2 S70C S16
Figure S6. LC-MS analyses of the OXA-48 S70C ampicillin products S17
Figure S7. Comparison of OXA-48 S70C products to a synthetic D-Phg-Gly dipeptide S18
Figure S8. NMR profiles of penicillin hydrolysis and fragmentation products S19
Table S6. Chemical shift assignments for faropenem S20
Table S7. Chemical shift assignments for the faropenem-derived hydrolysis product S20
Table S8. Chemical shift assignments for faropenem-derived 3-hydroxybutyrate S21
Table S9. Chemical shift assignments for the faropenem-derived thiazole S21
Figure S9. LC-MS analyses of the products of Ldt_{Mt2} and oxacillin S22
Figure S10. Products of KPC-2 and KPC-2 S70C with faropenem S24
Figure S11. Comparison of the products formed by Ldt_{Mt2} and OXA-48 S70C with faropenem S25
Figure S12. Plot of computationally calculated pK_a values vs. experimental pK_a values used to derive thioester enolate and ester enolate pK_a values S26
Table S10. Calculated ΔG values with experimentally and computationally derived pK_a values for model compounds S27
Table S11. Calculated ΔG and computationally derived pK_a values for models of the fragmentation-derived complexes. S28
Table S12. XYZ coordinates and corrected Gibbs energy of compounds optimized using M06-2X/6-31++g(d,p) in DMSO S29
Table S13. XYZ coordinates and quasi-harmonic corrected Gibbs energy of compounds optimized using M06-2X/6-31++G(d,p) in water S33
References S35

Experimental

General

β-Lactams were from Apollo Scientific Ltd. (ampicillin), ChemCruz (cefapirin), Fluka (cefotaxime), Glentham Life Sciences (ertapenem, meropenem, oxacillin, ticarcillin), Molekula (imipenem), MP Biomedicals (piperacillin), Selleckchem (faropenem), or TOKU-E (cephalothin).

Protein Production

The plasmid pNIC28-Bsa4-Ldt$_{Mt2}$ Δ1-55 was prepared by ligation independent cloning,[1] using a codon-optimised synthetic gene as template (GeneArt, Thermo Fisher Scientific), and transformed with *Escherichia coli* BL21(DE3). An overnight culture of *E. coli* BL21(DE3) pNIC28-Bsa4-Ldt$_{Mt2}$ Δ1-55 was grown at 37 °C, 180 rpm in 2TY media supplemented with 50 µg/mL kanamycin. This culture was used to inoculate (1 %) a larger 2TY culture (with 50 µg/mL kanamycin), which was grown at 37 °C, 180 rpm to an OD_{600} of 0.6. Isopropyl β-D-thiogalactopyranoside (IPTG) was then added to a final concentration of 0.5 mM, and the culture was incubated at 18 °C, 180 rpm overnight. The cells were harvested by centrifugation (11,000 × g, 10 min), and stored at -80 °C.

Cell pellets were resuspended in HisTrap Buffer A (50 mM Tris, pH 8, 500 mM NaCl, 20 mM imidazole) with added DNase I, and lysed by sonication (SONIC Vibra-Cell, 60 % amplitude). Lysates were centrifuged (32,000 × g, 30 min), clarified with a 0.45 µm filter, and loaded onto a 5 mL HisTrap column (GE Life Sciences) pre-equilibrated in HisTrap Buffer A. The column was washed with HisTrap Buffer A, then protein was eluted with a gradient running from 0 % to 100 % HisTrap Buffer B (50 mM Tris, pH 8, 500 mM NaCl, 500 mM imidazole). Fractions containing Ldt$_{Mt2}$ (as observed by SDS-PAGE) were combined, concentrated using a 30 kDa molecular weight cut-off (MWCO) Amicon centrifugal filter (EMD Millipore), and loaded onto a 300 mL

Superdex 200 column (GE Life Sciences) pre-equilibrated in gel filtration buffer (50 mM Tris, pH 8, 100 mM NaCl). Ldt_{Mt2} was eluted using gel filtration buffer; the desired fractions were combined and concentrated as above, and frozen using liquid nitrogen. The identity and purity of Ldt_{Mt2} was confirmed by mass spectrometry (calculated 40444 Da, observed $[M+H]^+$ 40441) and SDS-PAGE (>95% purity).

Plasmids encoding for Ldt_{Mt2} C354S, OXA-48 S70C and KPC-2 S70C were prepared by site-directed mutagenesis according to standard protocols, using pNIC28-Bsa4-Ldt_{Mt2} Δ1-55, pNIC28-Bsa4-OXA-48 and pOPINF-KPC-2 as templates, respectively. Ldt_{Mt2} C354S was purified as described above for Ldt_{Mt2}, while OXA-48 and KPC-2 were purified according to previously described methods;[2,3] the His-tag of KPC-2 was cleaved using 3C protease, while the His-tag of OXA-48 was not cleaved. The OXA-48 S70C and KPC-2 S70C variants were purified according to the same protocol as the wild-type enzymes (note that the His-tags were not removed). The identities and purities of the isolated proteins were confirmed by mass spectrometry (Ldt_{Mt2} C354S: calculated 40428 Da, observed $[M+H]^+$ 40420; OXA-48: calculated 30831 Da, observed $[M+H]^+$ 30832; OXA-48 S70C: calculated 30846 Da, observed $[M+H]^+$ 30847; KPC-2: calculated 28332 Da, observed $[M+H]^+$ 28330; KPC-2 S70C: calculated 30203 Da, observed $[M+H]^+$ 30201) and SDS-PAGE (>95% purity).

NMR Spectroscopy

NMR experiments were performed using a Bruker AVIIIHD 600 MHz spectrometer equipped with a Prodigy N2 broadband cryoprobe and a Bruker AVIII 700 MHz spectrometer equipped with an inverse TCI cryoprobe. Samples were prepared using 50 mM sodium phosphate, pH 7.5, 10 % D_2O. The water signal was suppressed using pre-saturation or excitation sculpting with perfect echo. β-Lactam degradation assays were performed using 2 mM substrate (ampicillin,

faropenem, oxacillin, piperacillin, ticarcillin) and enzyme (Ldt_{Mt2} – 10 μM; OXA-48 – 10 μM; OXA-48 S70C – 100 μM; KPC-2 – 1 μM; KPC-2 S70C – 10 μM). Chemical shift assignments were made on the basis of ^{1}H, COSY, HSQC, and HMBC spectra.

Mass Spectrometry

LC-MS experiments were performed using a Waters Xevo G2-S QTof mass spectrometer equipped with an Acquity UPLC system. An Aeris 3.6 μm WIDEPORE C4 200 Å LC column (50 × 2.1 mm; Phenomenex) was used, with solvents A (water with 0.1 % formic acid) and B (acetonitrile with 0.1 % formic acid) as mobile phases. The solvent gradient started at 5 % B for 1 min, then was increased to 95 % B over 5 min. Protein mass spectra were acquired using a Waters Micromass LCT Premier XE spectrometer equipped with an Acquity UPLC system. Protein spectra were deconvoluted using the MaxEnt 1 function in MassLynx 4.0 (Waters). Samples consisted of enzyme (1 μM) and substrate (100 μM) prepared in 50 mM sodium phosphate, pH 7.5.

Enzyme Kinetics

Kinetic assays were performed in, at least, triplicate, using 384-well black-walled microplates (Greiner Bio-One, PS, F-bottom, μCLEAR, black) and monitored on a BMG LABTECH PHERAstar FS instrument. The indicated concentrations of enzyme and fluorogenic substrate FC5[4] were prepared in 100 mM sodium phosphate, pH 7.5, 0.01 % Triton X-100. Fluorescence intensity was measured (λ_{ex} = 380 nm and λ_{em} = 460 nm) at 298 K every 2.5 min. Non-linear regression analysis was performed with Prism 5 (GraphPad Software).

Synthesis of the Ampicillin-Derived Dipeptide

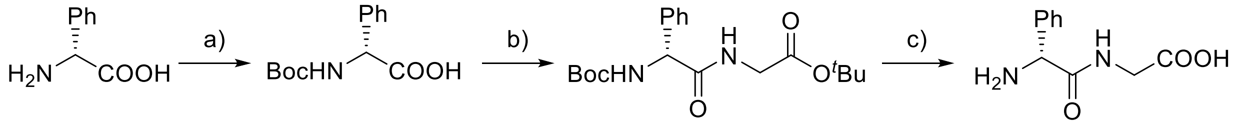

2 3 1

Scheme S1: Synthesis of fragment **1**: a) NaOH, Boc_2O, $^tBuOH/H_2O$; b) *i.* EDCI, Et_3N, *ii.* H-Gly-O^tBu; c) TFA, CH_2Cl_2.

Synthesis of (*S*)-2-((*tert*-butoxycarbonyl)amino)-2-phenylacetic acid 2

NaOH (635 mg, 15.9 mmol, 1.2 equivalents) was added to D-phenylglycine (2 g, 13.2 mmol, 1 equivalent) dissolved in $^tBuOH/H_2O$ (1:1, 15 mL), affording a clear colourless solution. To this solution, di-*tert*-butyl dicarbonate (3.17 g, 14.5 mmol, 1.1 equivalents) was added. The solution was stirred for 16 hours, before addition of EtOAc (40 mL), H_2O (30 mL), and HCl (1 M, 15 mL, 1.1 N, pH ~ 3). The aqueous layer was extracted with EtOAc (3 × 50 mL), and the combined organic extracts were washed with brine (100 mL), dried over Na_2SO_4, filtered and concentrated. The crude product was purified by column chromatography (25 g cartridge, EtOAc in c-hexane with 0.1% formic acid): 5% (3 CV) 5-30% EtOAc (over 20 CV), affording purified compound **2** as a clear colourless oil (2.5 g, 75%). $[\alpha]^{25}_D$ -119.8 (*c* 1.35, $CHCl_3$). FT-IR vmax (cm^{-1}): 3302, 2979, 2932, 2360, 1724, 1659, 1498, 1396, 1369, 1294, 1164, 1053, 699. ^1H NMR (600 MHz, Acetone-d_6) δ: 7.34 (d, J = 7.5 Hz, 2H), 7.24 (t, J = 7.5 Hz, 2H), 7.19 (t, J = 7.5 Hz, 1H), 6.40 (d, J = 5 Hz, 1H), 5.17 (bs, 1H), 1.27 (s, 9H). ^{13}C NMR (151 MHz, Acetone-d_6) δ: 171.6, 155.0, 137.8, 128.5, 128.0, 127.5, 78.6, 57.6, 27.7. HRMS (ESI^+) $C_{13}H_{17}O_4NNa$, ($[M+Na]^+$) requires 274.1055; found 274.1045.

Synthesis of *tert*-butyl (*S*)-(2-((*tert*-butoxycarbonyl)amino)-2-phenylacetyl)glycinate 3

Compound **2** (251 mg, 1 mmol, 1 equivalent), EDCI.HCl (170 mg, 1.1 mmol, 1.1 equivalents), and Et_3N (278 µL, 2 mmol, 2 equivalents) were dissolved in CH_2Cl_2 (5 mL). After stirring for 5 min, H-Gly-O^{t}Bu.HCl (167 mg, 1 mmol, 1 equivalent) was added. After stirring for 16 h, H_2O (15 mL) and CH_2Cl_2 (15 mL) were added. The aqueous layer was extracted with CH_2Cl_2 (2 × 20 µL). The combined organic extracts were dried over Na_2SO_4, filtered, and concentrated. The crude oil was purified by column chromatography (10 g cartridge, EtOAc in c-hexane): 0% (5 CV), 0-30% over 20 CV, affording purified compound **3** as a clear colourless oil (321 mg, 88%). $[\alpha]^{25}_D$ -89.7 (*c* 0.43, $CHCl_3$). FT-IR vmax (cm^{-1}): 3316, 2979, 2933, 2361, 2341, 1715, 1666, 1497, 1392, 1368, 1247, 1161, 1049, 698. ^{1}H NMR (600 MHz, $CDCl_3$) δ: 7.36 – 7.27 (m, 4H), 7.27 – 7.23 (m, 1H), 6.14 (s, 1H), 5.66 (s, 1H), 5.11 (s, 1H), 3.92 (dd, *J* = 18.5, 4.5 Hz, 1H), 3.76 (dd, *J* = 18.5, 4.5 Hz, 1H), 1.37 (s, 9H), 1.34 (s, 9H). ^{13}C NMR (151 MHz, $CDCl_3$) δ: 170.0, 168.4, 155.1, 138.2, 129.1, 128.4, 127.3, 82.6, 80.2, 58.7, 42.3, 28.3, 28.0. HRMS (ESI$^+$) $C_{15}H_{20}O_5N_2Na$, ($[M+Na]^+$) requires 331.1270; found 331.1254.

Synthesis of (*S*)-(2-amino-2-phenylacetyl)glycine 1

Compound **3** (321 mg) was dissolved in trifluoroacetic acid (TFA) (3 mL) and CH_2Cl_2 (3 mL), and stirred for 3 h, before coevaporating the TFA with CH_2Cl_2 (3 × 10 mL). The crude oil was purified by preparative scale HPLC, affording a white solid (t_r: 13 min). $[\alpha]^{25}_D$ -83.7 (*c* 1.07, MeOH). FT-IR vmax (cm^{-1}): 3070, 2938, 2637, 1668, 1539, 1192, 1140, 724, 699. ^{1}H NMR (600 MHz, D_2O) δ: 7.50 – 7.43 (m, 5H), 5.16 (s, 1H), 3.93 (dd, *J* = 18.0, 7.5 Hz, 2H). ^{13}C NMR (151 MHz, D_2O) δ: 173.1, 169.0, 131.6, 130.4, 129.6, 128.2, 56.6, 41.4. These NMR data are consistent

with the previously characterised enantiomer of **1**, i.e., (*R*)-(2-amino-2-phenylacetyl)glycine.[5] HRMS (ESI^+) $C_{10}H_{12}O_3N_2Na$, ($[M+Na]^+$) requires 231.0746; found 231.0734.

Computational Methodology

Density functional theory (DFT) calculations were performed with Gaussian09[6] and quasi-harmonic corrections were applied to the energy outputs using the GoodVibes script developed by Paton and Funes-Ardoiz.[7] A linear regression model (Figure S12) was calculated comparing experimental pK_a values[8] of 11 compounds against pK_a values obtained at the M06-2X/6-31++g(d,p) [9] level of theory with implicit polarizable continuum model (PCM) solvation[10] in DMSO. Following benchmarking with DMSO, the calculations were repeated in aqueous conditions. The pK_a values were calculated from ΔG for acid dissociation in solution where:

$$\Delta G = G\ (H^{+}) + G(A^{-}) - G(AH)$$

and

$$pKa = \Delta G/2.303RT$$

Values of -273.3 kcal/mol and -265.9 kcal/mol were used for the H^+ solvation term[11] in DMSO and water, respectively; the temperature was set at 298.15 K. A strong correlation coefficient was obtained (R^2 = 0.95, RMSE 1.8). This model (y = 1.028x – 0.733) was then used to calculate pK_a values of models of the predicted thioester and ester enolate fragmentation products of ampicillin and faropenem in DMSO. Once the DFT level of theory and basis set were benchmarked, they were applied to these systems in water.

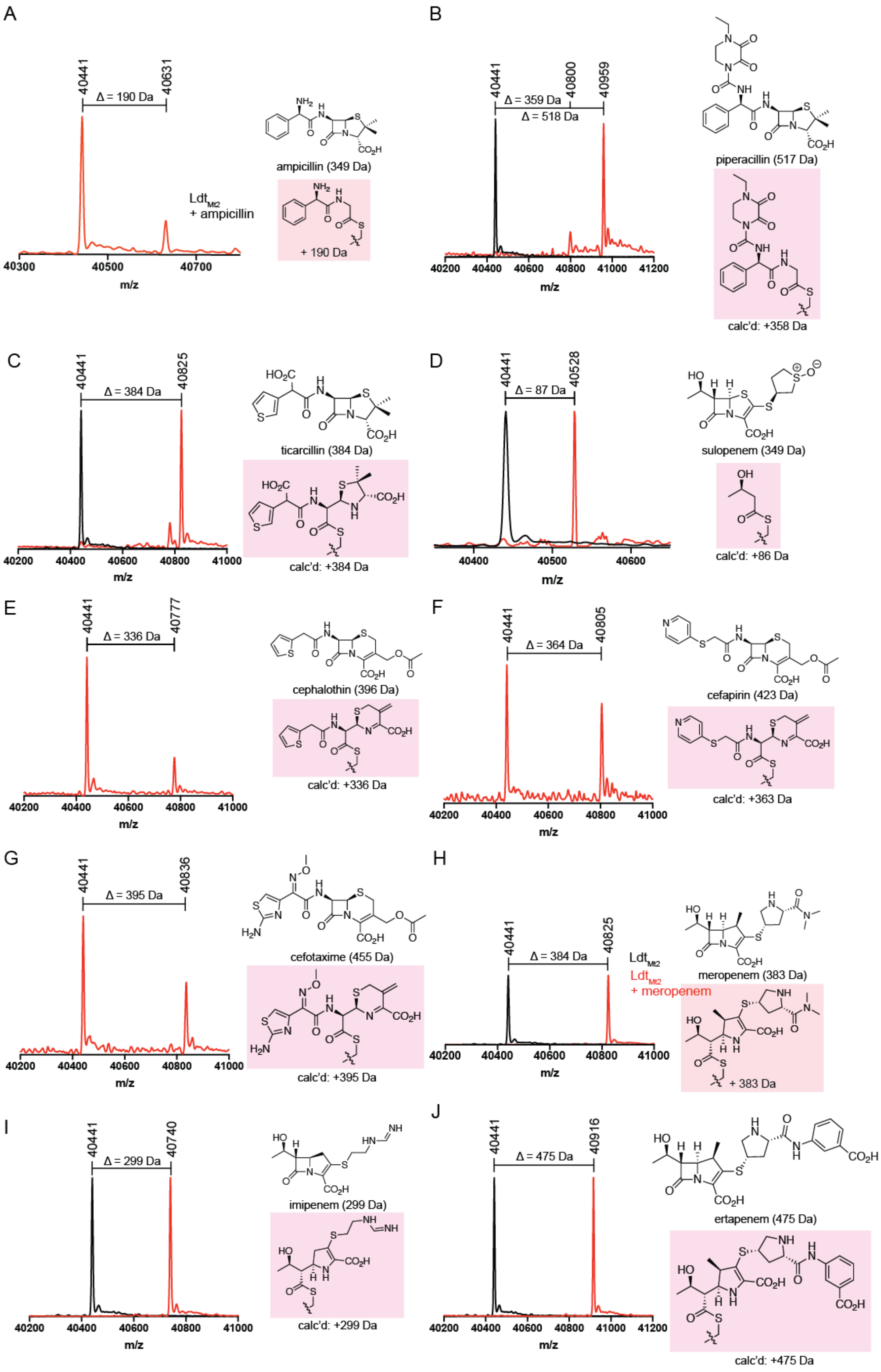
A
40441
40631
Δ = 190 Da
Ldt
Mt2
+ ampicillin
40300
40500
40700
m/z
ampicillin (349 Da)
+ 190 Da
B
40441
40800
40959
Δ = 359 Da
Δ = 518 Da
40200
40400
40600
40800
41000
41200
m/z
piperacillin (517 Da)
calc'd: +358 Da
C
40441
40825
Δ = 384 Da
40200
40400
40600
40800
41000
m/z
ticarcillin (384 Da)
calc'd: +384 Da
D
40441
40528
Δ = 87 Da
40400
40500
40600
m/z
sulopenem (349 Da)
calc'd: +86 Da
E
40441
40777
Δ = 336 Da
40200
40400
40600
40800
41000
m/z
cephalothin (396 Da)
calc'd: +336 Da
F
40441
40805
Δ = 364 Da
40200
40400
40600
40800
41000
m/z
cefapirin (423 Da)
calc'd: +363 Da
G
40441
40836
Δ = 395 Da
40200
40400
40600
40800
41000
m/z
cefotaxime (455 Da)
calc'd: +395 Da
H
40441
40825
Δ = 384 Da
Ldt
Mt2
Ldt
Mt2
+ meropenem
40200
40400
40600
40800
41000
m/z
meropenem (383 Da)
+ 383 Da
I
40441
40740
Δ = 299 Da
40200
40400
40600
40800
41000
m/z
imipenem (299 Da)
calc'd: +299 Da
J
40441
40916
Δ = 475 Da
40200
40400
40600
40800
41000
41200
m/z
ertapenem (475 Da)
calc'd: +475 Da

Figure S1. Mass spectra for the complexes derived from incubation of Ldt_{Mt2} with a panel of β-lactams. Purified Ldt_{Mt2} (1 μM) was treated with a 100-fold excess of (A) ampicillin for 140 min, (B) piperacillin for 2 h, (C) ticarcillin for 2 h, (D) sulopenem for 20 min, (E) cephalothin for 3 h, (F) cefapirin for 3 h, (G) cefotaxime for 3 h, (H) meropenem for 10 min, (I) imipenem for 30 min, and (J) ertapenem for 30 min. Samples were prepared in 50 mM sodium phosphate, pH 7.5. The black chromatograms represent Ldt_{Mt2} alone, while the red chromatograms represent the mixture of Ldt_{Mt2} with a β-lactam antibiotic. Proposed structures consistent with the observed mass shifts for the acylated products are highlighted in pink. Note that the tautomeric states/stereochemistries of the imipenem and ertapenem-derived pyrroline rings are not known.

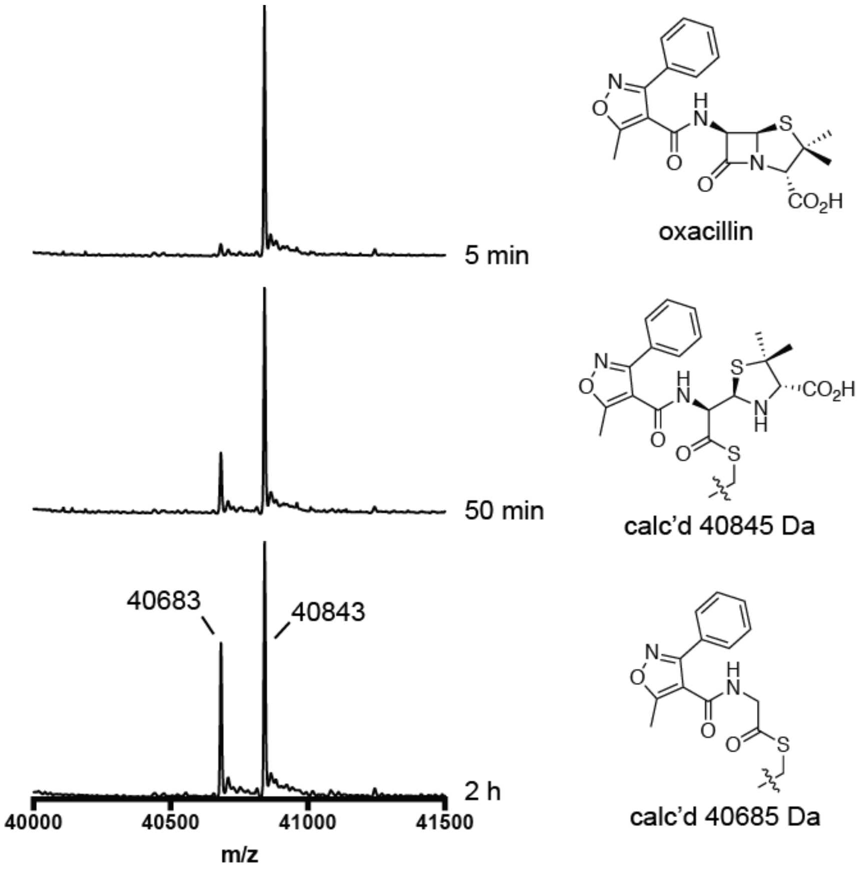

Figure S2. C5-C6 fragmentation of the oxacillin-derived Ldt_{Mt2} acyl-enzyme complex. Mass spectra showing the accumulation over time of Ldt_{Mt2} acylated with the oxacillin C5-C6 fragmentation product. Ldt_{Mt2} (1 μM) and oxacillin (100 μM) were prepared in 50 mM sodium phosphate, pH 7.5. The signals observed are consistent with the masses of the proposed adducts.

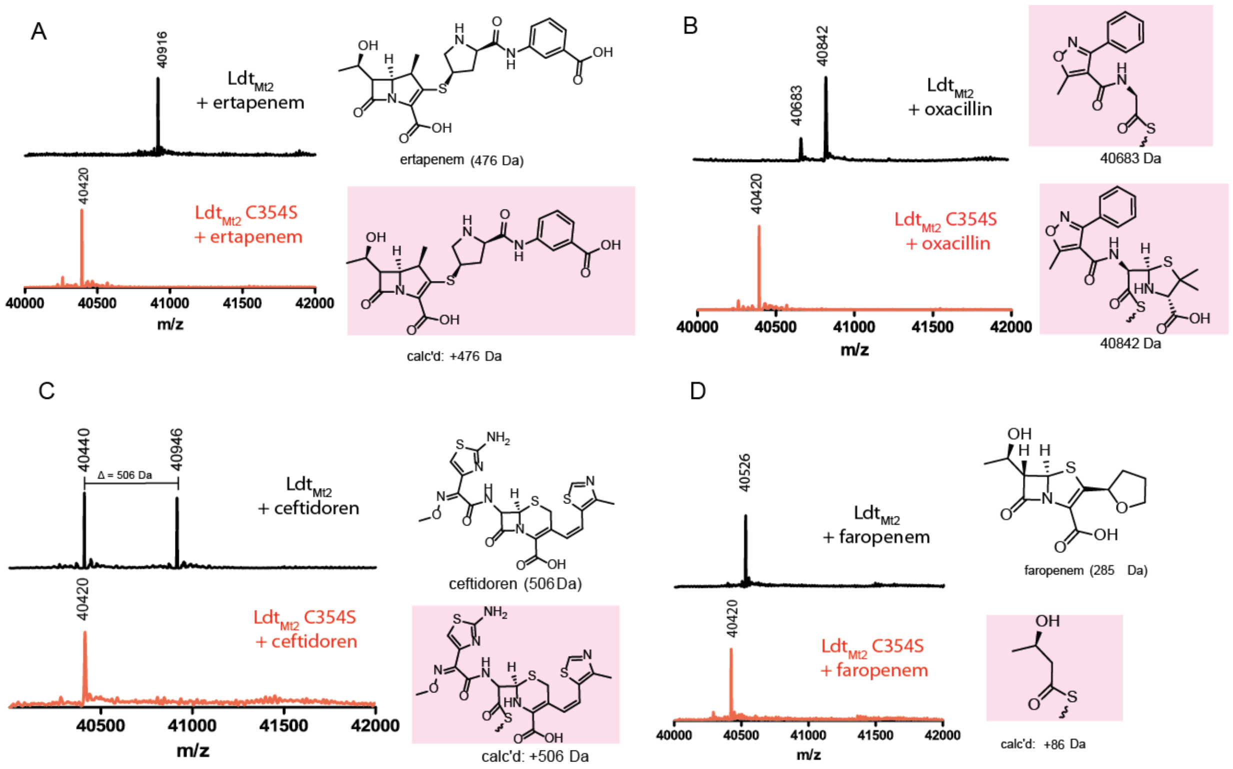

Figure S3. Characterization of Ldt$_{Mt2}$ C354S. Mass spectra showing the absence of acylation of Ldt$_{Mt2}$ C354S (2.2 µM), in comparison to wild-type Ldt$_{Mt2}$ (1.6 µM; ertapenem measured with 4.4 µM enzyme), upon treatment with (A) ertapenem (100 µM) for 10 min (wild-type) or 50 min (C354S variant), (B) oxacillin (100 µM) for 50 min (wild-type) or 70 min (C354S variant) (see Figure S2 for more detail), (C) ceftidoren (100 µM) for 5 min (both enzymes), and (D) faropenem (100 µM) for 20 min (wild-type) or 50 min (C354S variant). Samples were prepared in 50 mM sodium phosphate, pH 7.5. The proposed structures of the acyl-enzyme complexes are shown with a pink background. Wild-type Ldt$_{Mt2}$ in the absence of β-lactam was observed at 40440 Da, while Ldt$_{Mt2}$ C354S in the absence of β-lactam was observed at 40420 Da. Black spectra: wild-type Ldt$_{Mt2}$ with the indicated β-lactam; red spectra: Ldt$_{Mt2}$ C354S with the indicated β-lactam.

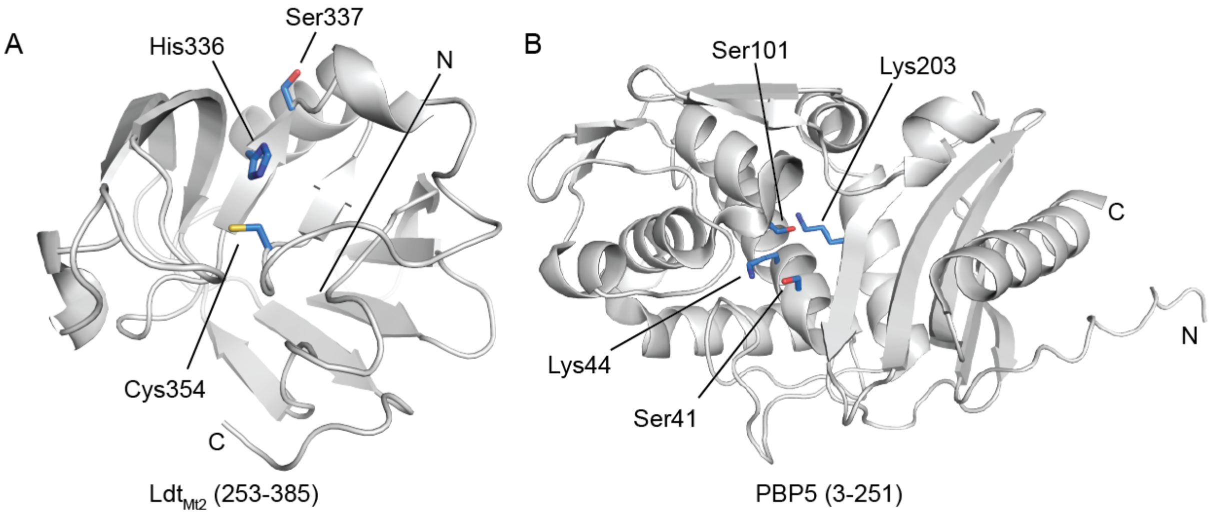

Figure S4. Comparison of the active sites of Ldt$_{Mt2}$ and PBP5. View of the active sites derived from crystal structures of (A) Ldt$_{Mt2}$ from *Mycobacterium tuberculosis* (PDB 3VYO)[12] and (B) PBP5 from *Pseudomonas aeruginosa* (PDB 4K91).[13] Active site residues, including the nucleophiles Cys354 (Ldt$_{Mt2}$) and Ser41 (PBP5), are shown in blue. The N- and C-termini are indicated by N and C, respectively.

Table S1. Michaelis-Menten kinetic constants for the enzyme-catalysed hydrolysis of FC5[4] by OXA-48 and OXA-48 S70C.

Enzyme	OXA-48	OXA-48 S70C
[E] / nM	5	25
K_M / μM	56 ± 6	6.8 ± 0.9
V_{max} / μM s^{-1}	0.100 ± 0.006	0.0167 ± 0.0007
k_{cat} / s^{-1}	20 ± 1	0.667 ± 0.029
k_{cat}/K_M / μM^{-1} s^{-1}	0.36 ± 0.06	0.098 ± 0.017

Table S2. Chemical shift assignments for ampicillin.

Position	^{13}C (ppm)	^{1}H (ppm)
1		
2	66.8	
3	76.1	4.17
4		
5	69.1	5.48
6	60.9	5.47
7	177.0	
8, 9	29.2, 33.0	1.44, 1.49
10	177.1	
11		
12		
13		
14		
15, 16, 17	130.4, 131.9	7.46 (m)

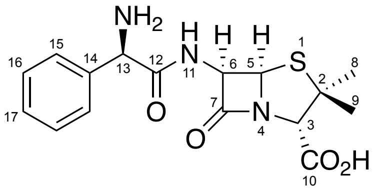

Table S3. Chemical shift assignments for the ampicillin-derived hydrolysis product.

Position	^{13}C (ppm)	^{1}H (ppm)
1		
2	61.4	
3	78.2	3.14
4		
5	68.5	5.07
6	62.6	4.26
7	178.9	
8, 9	28.8, 29.0	1.15, 1.24
10	178.1	
11		
12	174.7	
13	60.6	4.92
14	138.9	
15, 16, 17	130.1, 132.2	7.50 (m)

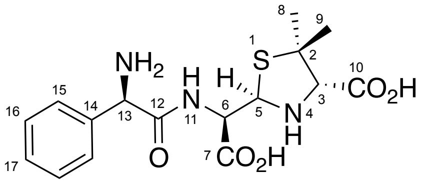

Table S4. Chemical shift assignments for the ampicillin-derived dipeptide.

Position	^{13}C (ppm)	^{1}H (ppm)
1	179.5	
2	46.1	3.67, 3.86
3		
4	174.0	
5	60.3	5.04
6	138.2	
7, 8, 9		

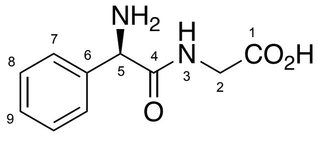

Table S5. Chemical shift assignments for the ampicillin-derived dihydrothiazole.

Position	^{13}C (ppm)	^{1}H (ppm)
1		
2	61.7	
3	89.8	4.50
4		
5	164.4	8.20
6, 7	28.0, 32.2	1.42, 1.65
8	178.6	

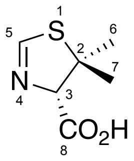

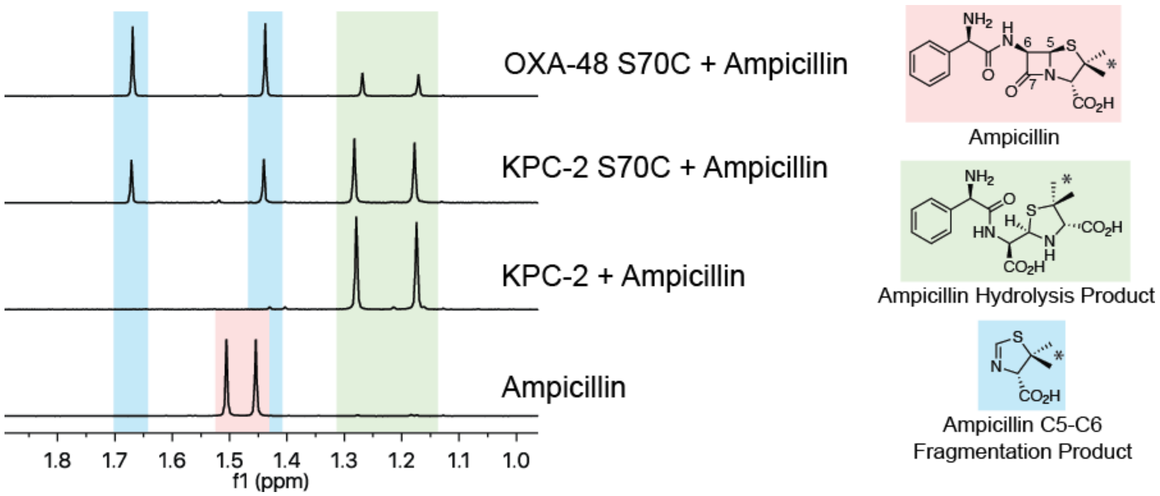

Figure S5. Degradation of ampicillin by KPC-2 and KPC-2 S70C. ^{1}H-NMR spectra (600 MHz) showing the methyl group resonances of the products formed by KPC-2 (1 μM), KPC-2 S70C (10 μM), and OXA-48 S70C (100 μM) with ampicillin (2 mM) in 50 mM sodium phosphate, pH 7.5, 10 % D_2O. Chemical shift assignments are listed in Table S2 (ampicillin), Table S3 (ampicillin-derived hydrolysis product), and Table S5 (ampicillin-derived dihydrothiazole product). These signals correspond to the methyl group protons indicated by asterisks in the structures on the right.

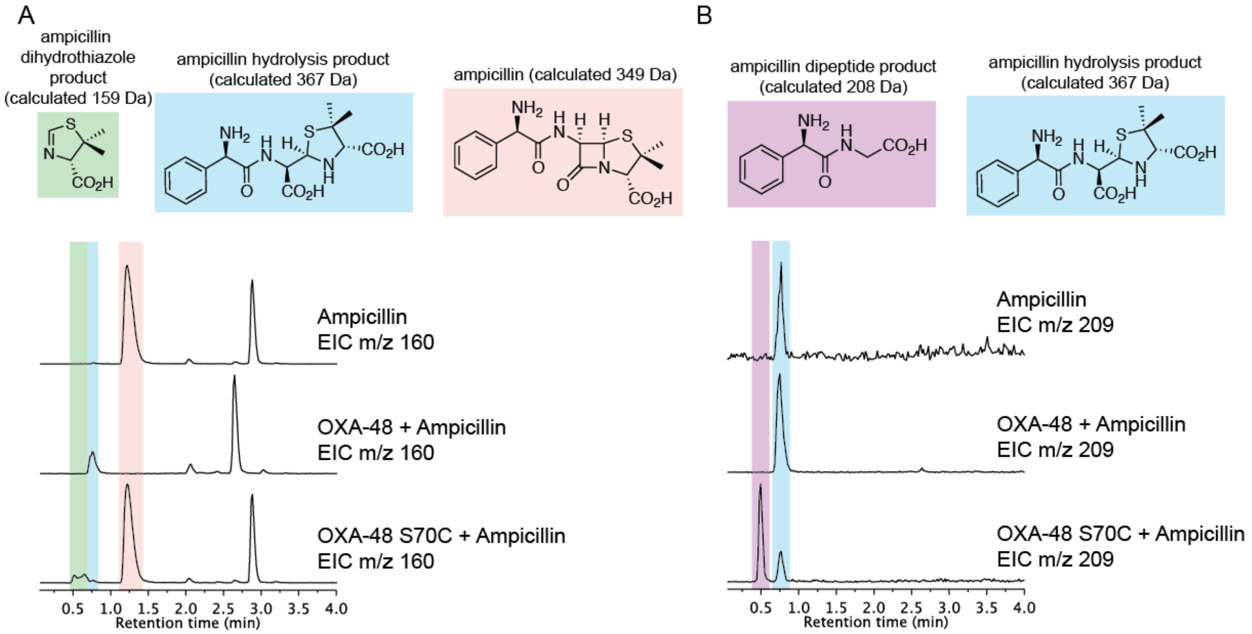

Figure S6. LC-MS analyses of the products formed by OXA-48 S70C with ampicillin. Extracted ion chromatograms at (A) m/z 160 and (B) m/z 209, for ampicillin, the mixture of OXA-48 with ampicillin, and the mixture of OXA-48 S70C with ampicillin. Peaks corresponding to the ampicillin-derived dihydrothiazole (calculated 159.04 Da, observed $[M+H]^+$ 160.05) and dipeptide (calculated 208.08, observed $[M+H]^+$ 209.10) products were observed for the mixture of OXA-48 S70C and ampicillin. In-source fragmentation of ampicillin yielded a signal at m/z 160, while in-source fragmentation of the ampicillin hydrolysis product (formed by OXA-48 and OXA-48 S70C) yielded peaks at m/z 160 and m/z 209. Samples consisted of 100 µM ampicillin and 1 µM enzyme in 50 mM sodium phosphate, pH 7.5, and were incubated for 10 min before injection onto the LC-MS. Chromatogram intensities are normalised to the intensity of the strongest peak.

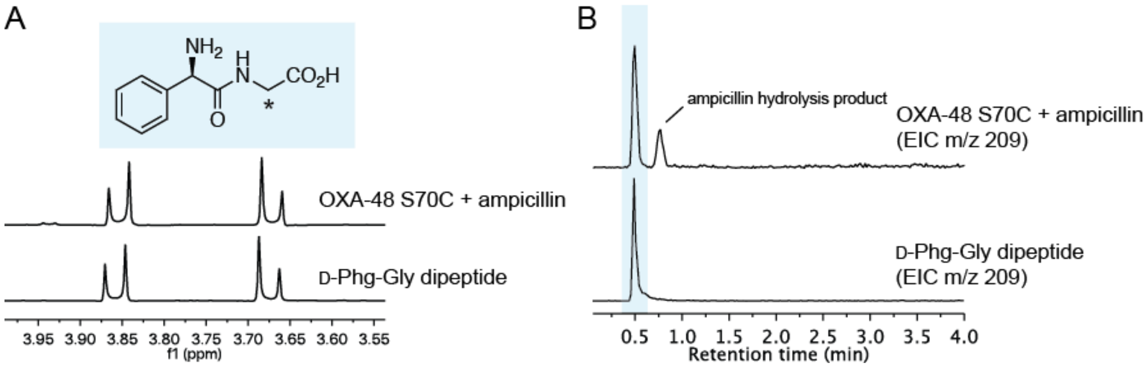

Figure S7. Comparison of OXA-48 S70C products with synthetic D-phenylglycine (Phg)-Gly dipeptide. (A) NMR spectra (700 MHz) comparing the synthetic D-Phg-Gly dipeptide to the ampicillin fragmentation product formed by OXA-48 S70C. The NMR signals correspond to the methylene position indicated with an asterisk in the chemical structure. The chemical shift assignments for the synthetic dipeptide are consistent with the assignments for the ampicillin-derived dipeptide (Table S4). (B) Extracted ion chromatograms (m/z 209) of the synthetic D-Phg-Gly dipeptide and the mixture of OXA-48 S70C with ampicillin.

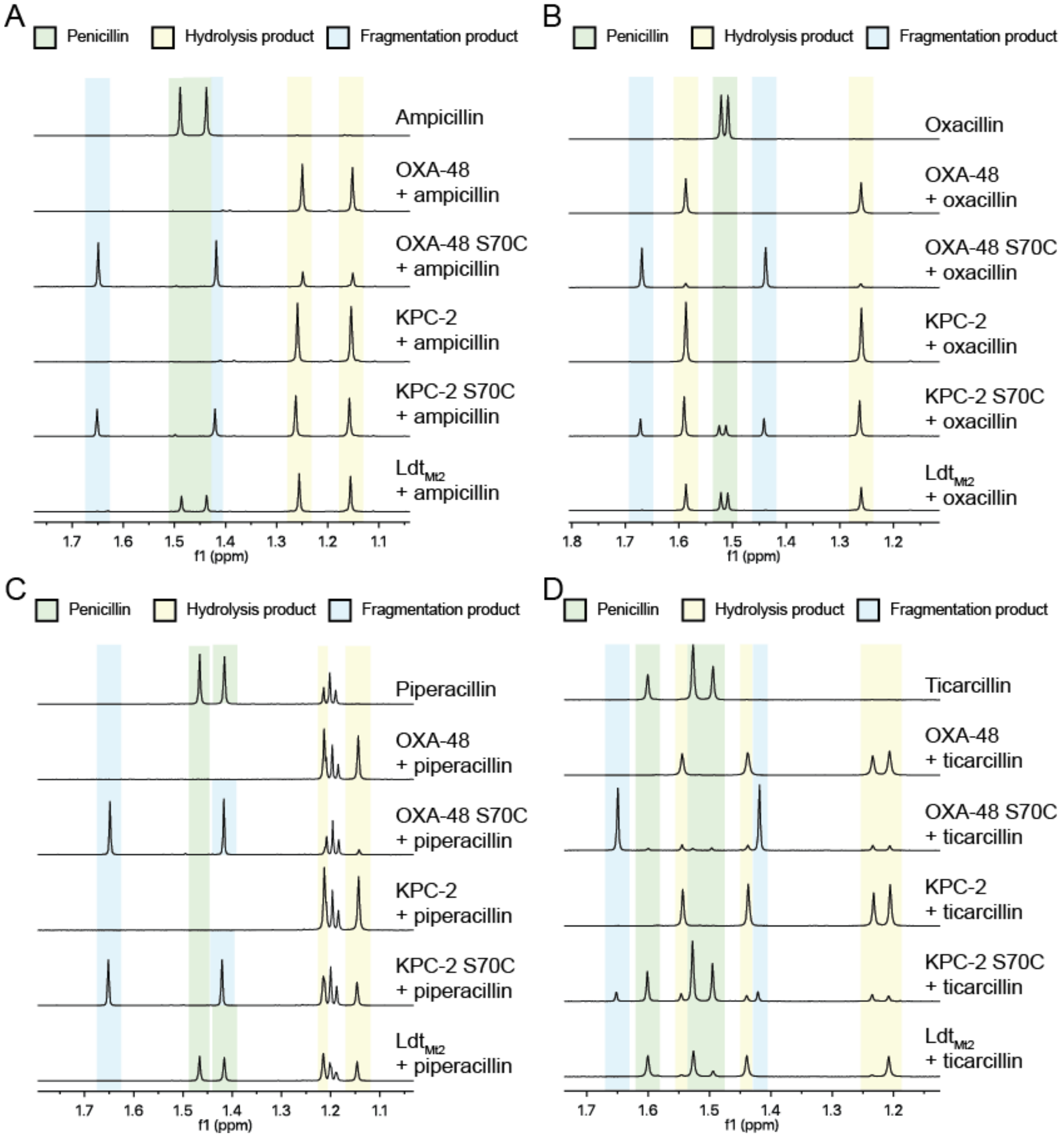

Figure S8. NMR profiles of penicillin hydrolysis and fragmentation products. ^{1}H NMR spectra (600 MHz) showing the products formed following the treatment of the penicillins (A) ampicillin, (B) oxacillin, (C) piperacillin, and (D) ticarcillin (all 2 mM) with OXA-48 (10 µM), OXA-48 S70C (100 µM), KPC-2 (1 µM), KPC-2 S70C (10 µM), and Ldt$_{Mt2}$ (10 µM). Samples were prepared in 50 mM sodium phosphate, pH 7.5, 10 % D_2O. See Figure S5 for the structures of ampicillin, the ampicillin hydrolysis product, and the ampicillin fragmentation products.

Table S6. Chemical shift assignments for faropenem.

Position	^{13}C (ppm)	^{1}H (ppm)
1		
2		
3		
4		
5	65.0	5.52
6	71.4	3.81
7	178.8	
8	67.7	4.17
9	22.9	1.24
10		
11	77.6	5.44
12	36.0	1.84, 2.31
13	28.5	1.89, 1.99
14	72.5	3.78, 3.88

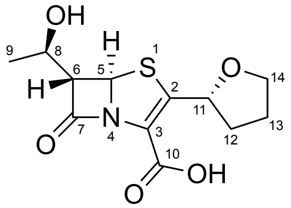

Table S7. Chemical shift assignments for the faropenem-derived hydrolysis products.

	Major Product[b]		Minor Product
Position[a]	^{13}C (ppm)	^{1}H (ppm)	^{1}H (ppm)
1			
2	142.1		
3	171.6		
4			
5	84.6	5.95	5.93
6	66.1	2.58	2.61
7	180.2		
8	71.2	4.18	4.07
9	22.2	1.20	1.20
10	181.5		
11	129.5	6.07	6.02
12	32.6	2.15	2.15
13	31.9	1.68	1.67
14	64.0	3.56	3.56

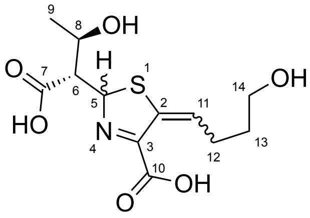

[a] Note that the stereochemistry of the exocyclic double bond is unclear based on these NMR data.
[b] Note that two sets of signals were observed for the faropenem hydrolysis product. The coupling between the protons on C-5 and C-6 differed between the major (J = 9.6 Hz) and minor (J = 7.6 Hz) products. Additionally, long range coupling between the C-5 and C-11 protons gave rise to similar coupling constants for the major (J = 1.4 Hz) and minor (J = 1.6 Hz) products. Based on these observations, we have assigned these two products as likely C-5 epimers.

Table S8. Chemical shift assignments for faropenem-derived 3-hydroxybutyrate.

Position	^{13}C (ppm)	^{1}H (ppm)
1	183.24	
2	49.4	2.31, 2.40
3	68.9	4.15
4	24.7	1.20

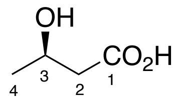

Table S9. Chemical shift assignments for the faropenem-derived thiazole.

Position	^{13}C (ppm)	^{1}H (ppm)
1		
2	154.8	8.77
3		
4		
5	149.5	
6		
7	78.1	5.76
8	38.3	1.87, 2.53
9	28.3	2.06
10	72.1	3.91, 4.11

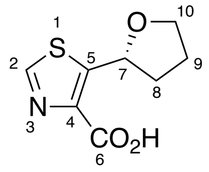

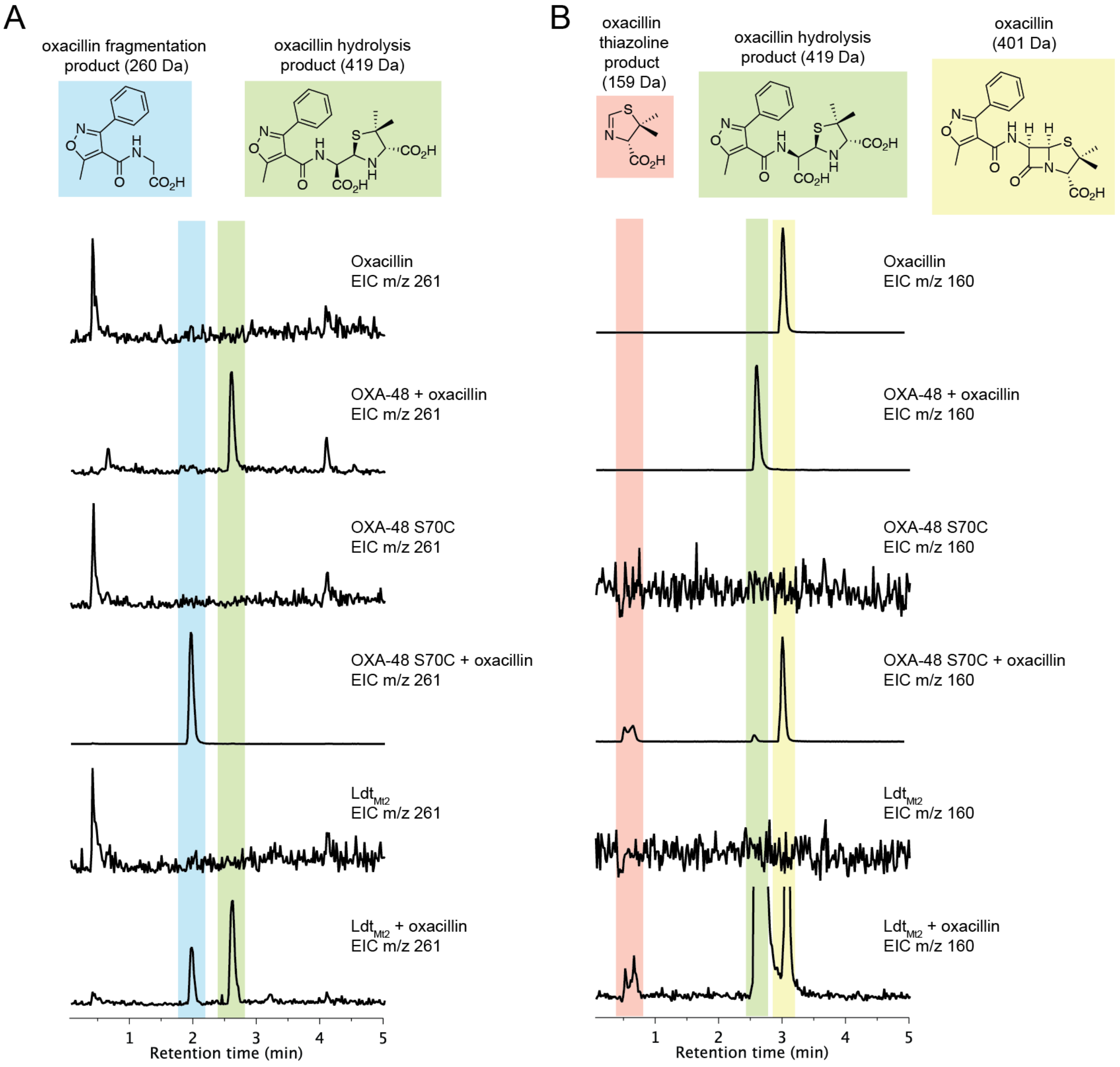

Figure S9. LC-MS analyses of the products of Ldt$_{Mt2}$ and oxacillin. Extracted ion chromatograms at (A) m/z 261 and (B) m/z 160, comparing the assigned products of OXA-48 (1 μM), OXA-48 S70C (1 μM) and Ldt$_{Mt2}$ (1 μM) with oxacillin (100 μM). Samples were prepared in 50 mM sodium phosphate, pH 7.5, and were incubated for 10 min (OXA-48, OXA-48 S70C) or 30 min (Ldt$_{Mt2}$) before injection. These results imply that OXA-48 S70C and Ldt$_{Mt2}$ form the same fragmentation products from oxacillin, i.e., an *N*-acylated glycine (m/z 261) and a dihydrothiazole (m/z 160). Note that in-source fragmentation was observed for oxacillin (m/z 160)

and the oxacillin-derived hydrolysis product (m/z 160, m/z 261). The intensities of the chromatograms are normalised according to the intensity of the most intense peaks (with the exception of the Ldt_{Mt2}/oxacillin m/z 160 extracted ion chromatogram).

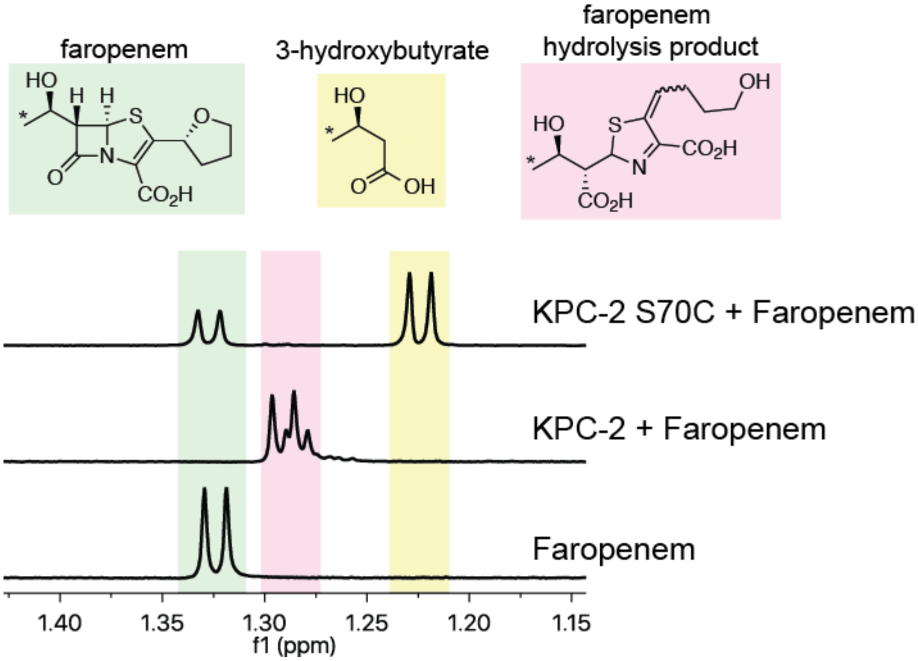

Figure S10. Products of KPC-2 and KPC-2 S70C with faropenem. ^{1}H-NMR spectra (600 MHz) showing the assigned products formed by KPC-2 (125 nM) and KPC-2 S70C (10 µM) with faropenem (1 mM) in 50 mM sodium phosphate, pH 7.5, 10 % D_2O. Chemical shift assignments are listed in Table S6 (faropenem), Table S7 (faropenem-derived hydrolysis product), and Table S8 (faropenem-derived 3-hydroxybutyrate). The peaks correspond to the methyl groups indicated with asterisks on the chemical structures. Note that evidence for epimerisation was observed of the faropenem-derived hydrolysis product (see Table S7).

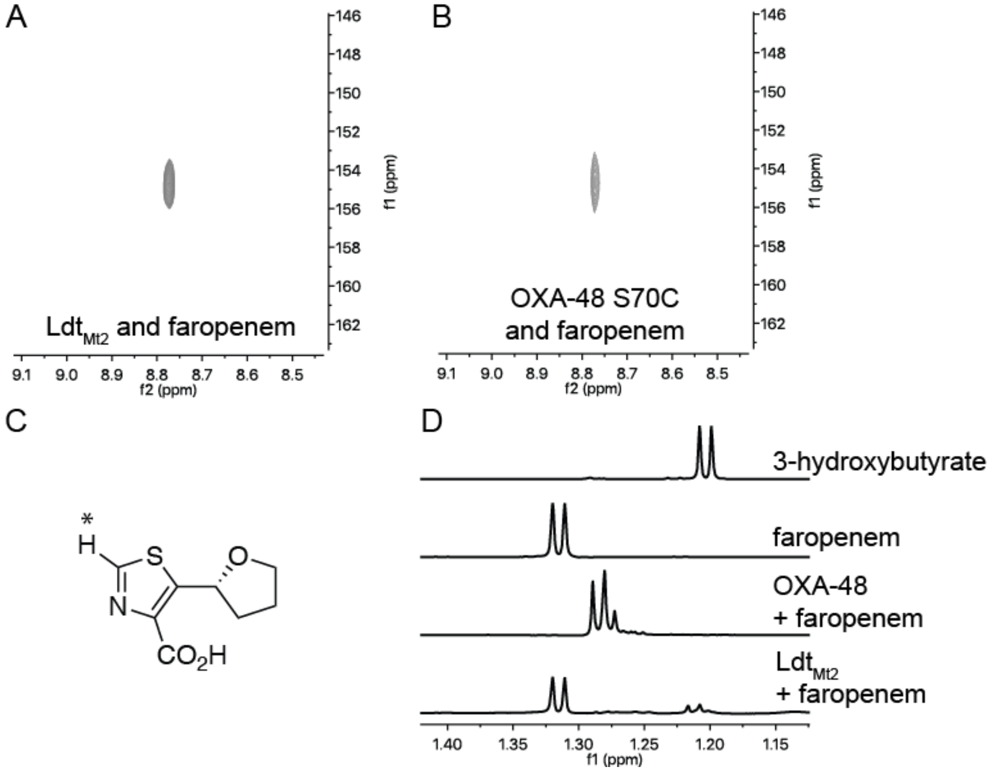

Figure S11. Comparison of ^{1}H-^{13}C-HSQC NMR spectra for the products formed by Ldt$_{Mt2}$ and OXA-48 S70C with faropenem. ^{1}H-^{13}C-HSQC spectra (700 MHz) showing the thiazole product formed by (A) Ldt$_{Mt2}$ with faropenem, and (B) OXA-48 S70C with faropenem. (C) Structure of the faropenem-derived thiazole product. The correlation indicated in panels A and B corresponds to the proton and carbon indicated with asterisks. (D) ^{1}H NMR spectra (600 MHz) comparing the products formed by Ldt$_{Mt2}$ (100 μM) and OXA-48 (2.5 μM) with faropenem (1 mM). After 20 h incubation of Ldt$_{Mt2}$ with faropenem, signals corresponding to 3-hydroxybutyrate were observed. Low levels of the hydrolysis product could also be observed in this sample, based on a comparison to the spectrum of OXA-48 and faropenem; these signals (in the spectrum showing Ldt$_{Mt2}$ and faropenem) likely result from non-enzymatic faropenem hydrolysis.

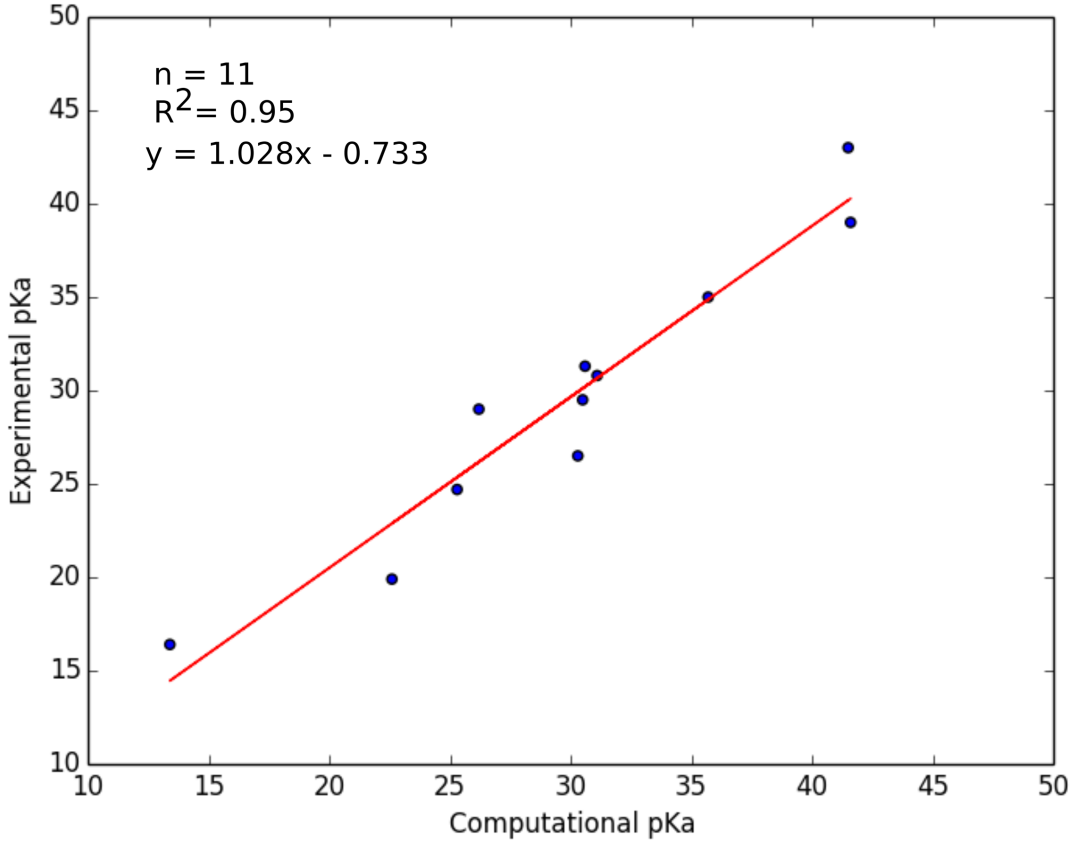

Figure S12. Plot of computationally calculated pK_a values vs. experimental pK_a values used to derive thioester enolate and ester enolate pK_a values. The experimental pK_a values are from Zhang et al.[8]

Table S10. Calculated ΔG values with experimentally and computationally derived pK_a values for model compounds.

Compound	ΔG (kcal/mol)[a]	Expt. pK_a[b]	Comp. pK_a
$PhSCH_3$	56.7	39	41.6
CH_3CN	41.6	31.3	30.6
$PhCOCH_3$	34.5	24.7	25.3
CH_3CONEt_2	48.6	35	35.7
CH_3CO_2Et	41.6	29.5	30.5
CH_3COCH_3	37.3	26.5	27.3
$PhSO_2CH_3$	35.6	29.0	26.2
$PhCH_3$	56.6	43.0	41.5
$CH_2(CO_2Et)_2$	18.3	16.4	13.4
$PhCH_2SPh$	42.4	30.8	31.1
$PhCH_2COCH_3$	30.8	19.9	22.6

a. M06-2X/6-31++g(d,p) in DMSO.
b. See Zhang et al.[8]

Table S11. Calculated ΔG and computationally derived pK_a values for models of the fragmentation-derived complexes. Values were obtained using the model shown in Figure S12. DFT calculations were performed in DMSO and water for the ampicillin thioester (CAmp) and ester (SAmp) enolate models and faropenem thioester (CFaro) and ester (SFaro) enolate models (in which the cysteine and serine side chains were replaced with methyl groups).

	DMSO			Water	
Compound	ΔG (kcal/mol)[a]	Comp. pK_a	Extrapol. pK_a	ΔG (kcal/mol)[b]	Comp. pK_a[c]
CAmp	24.8	18.2	19.5	35.2	23.3
SAmp	34.5	25.3	26.7	45.1	30.5
CFaro	31.7	23.3	24.7	42.1	28.3
SFaro	40.5	29.7	31.2	50.8	34.7

[a]M06-2X/6-31++g(d,p) in DMSO.
[b]M06-2X/6-31++g(d,p) in water.
[c]RMSE = 1.8.

Table S12. XYZ coordinates and quasi-harmonic corrected Gibbs energy of compounds optimized using M06-2X/6-31++g(d,p) in DMSO.

$CH_2(CO_2Et)_2$

E = -574.527408 a.u.

Atom	X	Y	Z
C	4.468	-0.548	-0.378
C	3.034	-0.17	-0.672
H	5.056	-0.481	-1.296
H	4.899	0.129	0.363
H	4.526	-1.572	-0.001
H	2.965	0.858	-1.036
H	2.593	-0.847	-1.41
O	2.304	-0.288	0.569
C	1.013	0.037	0.657
O	0.438	-0.093	1.715
C	0.331	0.57	-0.59
H	0.808	1.504	-0.899
H	0.419	-0.155	-1.404
C	-1.123	0.845	-0.283
O	-1.558	1.932	0.028
O	-1.863	-0.256	-0.386
C	-3.263	-0.119	-0.061
C	-3.896	-1.483	-0.209
H	-3.702	0.615	-0.741
H	-3.342	0.264	0.96
H	-4.961	-1.417	0.025
H	-3.789	-1.851	-1.233
H	-3.435	-2.201	0.474

$PhCH_2SPh$

E = -900.401501 a.u.

Atom	X	Y	Z
C	4.064	-1.218	0.247
C	2.693	-1.153	0.489
C	2.016	0.068	0.419
C	2.731	1.225	0.092
C	4.103	1.163	-0.151
C	4.772	-0.059	-0.074
H	4.58	-2.171	0.311
H	2.141	-2.056	0.74
H	2.21	2.177	0.033
H	4.649	2.068	-0.398
H	5.84	-0.107	-0.262
C	0.531	0.128	0.651
H	0.205	-0.661	1.333
H	0.234	1.099	1.054
S	-0.334	-0.12	-0.956
C	-2.03	-0.044	-0.395
C	-2.608	1.187	-0.066
C	-2.784	-1.216	-0.291
C	-3.928	1.24	0.378
H	-2.024	2.098	-0.159
C	-4.109	-1.157	0.142
H	-2.331	-2.169	-0.545
C	-4.681	0.069	0.48
H	-4.371	2.197	0.636
H	-4.691	-2.07	0.217
H	-5.711	0.113	0.82

$PhCH_2COCH_3$

E = -423.914283 a.u.

Atom	X	Y	Z
C	2.303	1.211	0.215
C	0.971	1.233	-0.196
C	0.3	0.046	-0.503
C	0.988	-1.166	-0.393
C	2.321	-1.193	0.017
C	2.982	-0.003	0.323
H	2.812	2.142	0.447
H	0.446	2.182	-0.281
H	0.476	-2.095	-0.632
H	2.842	-2.142	0.096
H	4.02	-0.021	0.64
C	-1.148	0.069	-0.911
H	-1.375	-0.752	-1.603
H	-1.397	0.992	-1.448
C	-2.126	-0.062	0.252
O	-1.758	-0.29	1.388
C	-3.583	0.114	-0.096
H	-4.209	-0.18	0.746
H	-3.764	1.167	-0.334
H	-3.841	-0.469	-0.984

CAmp

E = -1085.286470 a.u.

Atom	X	Y	Z
C	0.002	1.213	1.262
C	1.082	1.128	0.383
C	1.098	1.939	-0.758
C	0.042	2.804	-1.026
C	-1.044	2.876	-0.15
C	-1.057	2.083	0.995
H	-0.024	0.591	2.152
H	1.942	1.883	-1.441
H	0.065	3.424	-1.917
H	-1.869	3.55	-0.358
H	-1.896	2.132	1.683
C	2.247	0.172	0.608
H	3.173	0.736	0.439
N	1.369	-1.894	-0.508
H	1.44	-2.496	-1.32
C	0.154	-1.982	0.263
H	-0.112	-3.036	0.396
H	0.316	-1.557	1.256
S	-2.527	-1.365	0.545
C	-3.58	-0.342	-0.514
H	-4.546	-0.269	-0.015
H	-3.137	0.65	-0.619
H	-3.698	-0.807	-1.492
N	2.201	-0.46	1.924
H	3.02	-1.049	2.048
H	2.257	0.258	2.643
C	2.271	-0.88	-0.514
O	3.124	-0.801	-1.396
C	-1.024	-1.275	-0.406
O	-0.954	-0.712	-1.472

SAmp

E = -762.335973 a.u.

Atom	X	Y	Z
C	-2.012	-0.478	1.124
C	-2.081	0.397	0.033
C	-2.955	0.110	-1.017
C	-3.752	-1.037	-0.983
C	-3.678	-1.904	0.105
C	-2.805	-1.622	1.159
H	-1.336	-0.250	1.944
H	-3.016	0.787	-1.864
H	-4.431	-1.247	-1.804
H	-4.297	-2.795	0.134
H	-2.745	-2.295	2.009
C	-1.193	1.623	-0.021
H	-1.510	2.229	-0.887
N	0.521	0.193	-1.063
H	-0.227	-0.461	-1.247
C	1.876	-0.160	-1.390
H	1.872	-0.989	-2.101
H	2.393	0.683	-1.855
C	4.831	-1.023	0.641
H	5.843	-0.949	0.250
H	4.705	-0.372	1.508
H	4.597	-2.053	0.914
N	-1.231	2.365	1.234
H	-0.510	3.082	1.212
H	-2.129	2.832	1.324
C	0.278	1.291	-0.316
O	1.182	2.038	0.054
C	2.666	-0.591	-0.163
O	2.186	-0.915	0.899
O	3.972	-0.590	-0.425

CFaro

E = -744.918819 a.u.

Atom	X	Y	Z
C	0.873	0.937	0.311
C	1.742	-0.154	-0.302
C	3.201	0.277	-0.360
O	1.581	-1.308	0.520
C	-0.628	0.808	0.130
O	-1.348	1.786	0.092
H	1.139	1.914	-0.100
H	1.383	-0.376	-1.318
H	3.325	1.138	-1.022
H	3.554	0.546	0.641
H	3.822	-0.539	-0.741
H	1.984	-2.067	0.080
H	1.069	0.970	1.392
S	-1.304	-0.835	-0.005
C	-3.041	-0.386	-0.253
H	-3.151	0.220	-1.153
H	-3.587	-1.323	-0.366
H	-3.417	0.163	0.610

SFaro

E = -421.964707 a.u.

Atom	X	Y	Z
C	-0.481	-0.837	0.043
C	-1.630	0.124	-0.223
C	-2.980	-0.522	0.049
O	-1.406	1.240	0.638
C	0.845	-0.207	-0.305
O	1.064	0.420	-1.321
H	-0.595	-1.730	-0.582
H	-1.575	0.455	-1.268
H	-3.145	-1.369	-0.623
H	-3.029	-0.877	1.083
H	-3.787	0.200	-0.109
H	-2.051	1.928	0.434
O	1.777	-0.429	0.627
H	-0.482	-1.149	1.090
C	3.079	0.108	0.356
H	3.022	1.194	0.260
H	3.694	-0.170	1.208
H	3.478	-0.321	-0.564

$PhSCH_3$

E = -669.508004 a.u.

Atom	X	Y	Z
C	-1.833	1.275	-0.089
C	-0.466	1.131	-0.321
C	0.128	-0.133	-0.245
C	-0.657	-1.251	0.050
C	-2.027	-1.105	0.267
C	-2.616	0.157	0.203
H	-2.287	2.260	-0.149
H	0.141	1.998	-0.567
H	-0.193	-2.231	0.112
H	-2.632	-1.977	0.495
H	-3.681	0.271	0.376
S	1.878	-0.332	-0.552
C	2.552	0.455	0.946
H	2.215	-0.076	1.837
H	3.639	0.390	0.874
H	2.258	1.504	0.997

CH_3CN

E = -132.686570 a.u.

Atom	X	Y	Z
C	0.000	0.000	0.280
N	0.000	0.000	1.436
C	0.000	0.000	-1.182
H	0.000	1.028	-1.548
H	0.890	-0.514	-1.548
H	-0.890	-0.514	-1.548

$PhCOCH_3$

E = -384.645468 a.u.

Atom	X	Y	Z
C	-1.966	-1.135	0.000
C	-0.579	-1.224	0.000
C	0.204	-0.062	0.000
C	-0.421	1.190	-0.000
C	-1.812	1.279	-0.000
C	-2.584	0.118	-0.000
H	-2.568	-2.038	0.000
H	-0.082	-2.189	0.000
H	0.169	2.101	-0.000
H	-2.291	2.252	-0.000
H	-3.668	0.188	-0.000
C	1.696	-0.201	-0.000
O	2.212	-1.308	-0.000
C	2.544	1.047	0.000
H	3.595	0.764	-0.000
H	2.327	1.657	-0.882
H	2.328	1.656	0.883

CH_3CONEt_2

E = -366.161128 a.u.

Atom	X	Y	Z
C	2.136	-0.959	-0.300
H	1.680	-1.640	-1.023
H	2.372	-1.529	0.603
H	3.060	-0.559	-0.714
C	1.230	0.213	0.021
O	1.649	1.374	-0.078
N	-0.039	-0.044	0.419
C	-0.594	-1.387	0.582
C	-1.574	-1.765	-0.524
H	-1.095	-1.422	1.556
H	0.223	-2.106	0.625
H	-1.929	-2.788	-0.376
H	-2.444	-1.103	-0.524
H	-1.091	-1.700	-1.505
C	-0.925	1.092	0.681
C	-1.424	1.771	-0.592
H	-0.383	1.813	1.299
H	-1.767	0.713	1.268
H	-2.067	2.618	-0.337
H	-0.579	2.143	-1.176
H	-1.999	1.078	-1.211

CH_3CO_2Et

E = -307.502556 a.u.

Atom	X	Y	Z
C	2.133	-0.885	0.001
H	1.994	-1.584	-0.827
H	2.090	-1.457	0.931
H	3.097	-0.388	-0.083
C	1.034	0.138	-0.004
O	1.195	1.342	-0.003
O	-0.176	-0.434	-0.001
C	-1.311	0.455	0.008
C	-2.556	-0.404	-0.004
H	-1.253	1.103	-0.870
H	-1.255	1.081	0.903
H	-3.442	0.236	0.003
H	-2.587	-1.027	-0.901
H	-2.589	-1.050	0.876

CH_3COCH_3

E = -193.022725 a.u.

Atom	X	Y	Z
C	-0.000	0.179	0.000
O	-0.000	1.398	-0.000
C	1.284	-0.612	-0.003
H	2.137	0.047	-0.169
H	1.393	-1.114	0.964
H	1.250	-1.392	-0.769
C	-1.284	-0.612	0.003
H	-1.393	-1.114	-0.965
H	-1.250	-1.392	0.769
H	-2.137	0.047	0.169

$PhSO_2CH_3$

E = -819.843320 a.u.

Atom	X	Y	Z
C	-2.431	-1.157	0.000
C	-1.041	-1.203	0.000
C	-0.326	-0.004	0.000
C	-0.968	1.231	-0.000
C	-2.363	1.262	-0.000
C	-3.091	0.074	0.000
H	-3.000	-2.081	0.000
H	-0.518	-2.155	0.000
H	-0.411	2.161	-0.000
H	-2.876	2.217	-0.000
H	-4.175	0.105	0.000
S	1.451	-0.164	-0.000
O	1.849	-0.820	1.255
O	1.849	-0.820	-1.255
C	2.116	1.487	0.000
H	1.804	2.007	-0.905
H	1.804	2.007	0.905
H	3.198	1.344	0.000

$PhCH_3$

E = -271.355550 a.u.

Atom	X	Y	Z
C	-1.201	1.204	0.002
C	0.194	1.203	-0.009
C	0.913	0.002	-0.011
C	0.197	-1.201	-0.009
C	-1.197	-1.206	0.002
C	-1.902	-0.002	0.008
H	-1.740	2.147	0.002
H	0.734	2.146	-0.017
H	0.740	-2.143	-0.017
H	-1.734	-2.150	0.002
H	-2.988	-0.003	0.013
C	2.421	0.001	0.009
H	2.795	-0.103	1.033
H	2.823	-0.832	-0.574
H	2.822	0.932	-0.398

Table S13. XYZ coordinates and quasi-harmonic corrected Gibbs energy of compounds optimized using M06-2X/6-31++G(d,p) in water.

CAmp
E = -1085.286773a.u.

Atom	X	Y	Z
C	0.001	1.213	1.263
C	1.080	1.129	0.383
C	1.092	1.938	-0.759
C	0.035	2.800	-1.028
C	-1.050	2.872	-0.150
C	-1.061	2.081	0.996
H	-0.024	0.593	2.154
H	1.936	1.882	-1.444
H	0.055	3.418	-1.921
H	-1.877	3.544	-0.359
H	-1.899	2.130	1.685
C	2.247	0.176	0.608
H	3.171	0.742	0.439
N	1.372	-1.891	-0.508
H	1.444	-2.492	-1.321
C	0.157	-1.981	0.263
H	-0.107	-3.035	0.395
H	0.320	-1.556	1.256
S	-2.524	-1.368	0.545
C	-3.579	-0.346	-0.513
H	-4.545	-0.275	-0.014
H	-3.136	0.645	-0.619
H	-3.697	-0.812	-1.491
N	2.203	-0.456	1.924
H	3.023	-1.043	2.048
H	2.257	0.262	2.643
C	2.274	-0.876	-0.513
O	3.127	-0.797	-1.395
C	-1.022	-1.275	-0.406
O	-0.953	-0.712	-1.472

SAmp
E = -762.336314 a.u.

Atom	X	Y	Z
C	-2.003	-0.486	1.120
C	-2.079	0.395	0.035
C	-2.957	0.112	-1.013
C	-3.751	-1.037	-0.982
C	-3.670	-1.910	0.100
C	-2.793	-1.632	1.153
H	-1.323	-0.261	1.938
H	-3.024	0.795	-1.856
H	-4.433	-1.244	-1.800
H	-4.287	-2.803	0.128
H	-2.727	-2.310	1.998
C	-1.194	1.624	-0.016
H	-1.512	2.232	-0.880
N	0.522	0.207	-1.069
H	-0.224	-0.448	-1.257
C	1.879	-0.144	-1.394
H	1.877	-0.965	-2.115
H	2.398	0.704	-1.846
C	4.823	-1.031	0.643
H	5.837	-0.948	0.260
H	4.688	-0.393	1.518
H	4.590	-2.065	0.900
N	-1.236	2.363	1.241
H	-0.514	3.080	1.223
H	-2.133	2.829	1.331
C	0.277	1.297	-0.311
O	1.180	2.041	0.068
C	2.662	-0.591	-0.168
O	2.177	-0.929	0.887
O	3.970	-0.585	-0.423

CFaro
E = -744.918988 a.u.

Atom	X	Y	Z
C	0.873	0.936	0.312
C	1.742	-0.154	-0.302
C	3.201	0.277	-0.360
O	1.581	-1.308	0.519
C	-0.628	0.808	0.131
O	-1.347	1.786	0.092
H	1.139	1.914	-0.097
H	1.383	-0.375	-1.318
H	3.324	1.139	-1.021
H	3.555	0.545	0.641
H	3.822	-0.538	-0.742
H	1.984	-2.067	0.079
H	1.069	0.968	1.393
S	-1.304	-0.835	-0.004
C	-3.041	-0.386	-0.254
H	-3.151	0.220	-1.154
H	-3.587	-1.322	-0.367
H	-3.417	0.163	0.610

SFaro

E = -421.964896 a.u.

Atom	X	Y	Z
C	-0.489	-0.810	0.268
C	-1.622	0.066	-0.249
C	-2.983	-0.450	0.193
O	-1.367	1.371	0.269
C	0.843	-0.315	-0.240
O	1.078	-0.049	-1.401
H	-0.623	-1.835	-0.095
H	-1.569	0.093	-1.346
H	-3.168	-1.448	-0.216
H	-3.032	-0.502	1.285
H	-3.776	0.215	-0.160
H	-2.012	1.986	-0.101
O	1.755	-0.209	0.730
H	-0.492	-0.826	1.360
C	3.055	0.239	0.324
H	2.986	1.234	-0.118
H	3.654	0.263	1.232
H	3.482	-0.456	-0.400

References

[1] O. Gileadi, N. A. Burgess-Brown, S. M. Colebrook, G. Berridge, P. Savitsky, C. E. Smee, P. Loppnau, C. Johansson, E. Salah, N. H. Pantic, *Methods Mol Biol* **2008**, *426*, 221.

[2] S. T. Cahill, R. Cain, D. Y. Wang, C. T. Lohans, D. W. Wareham, H. P. Oswin, J. Mohammed, J. Spencer, C. W. Fishwick, M. A. McDonough, C. J. Schofield, J. Brem, *Antimicrob Agents Chemother* **2017**, *61*, e02260.

[3] O. A. Pemberton, X. Zhang, Y. Chen, *J Med Chem* **2017**, *60*, 3525.

[4] S. S. van Berkel, J. Brem, A. M. Rydzik, R. Salimraj, R. Cain, A. Verma, R. J. Owens, C. W. Fishwick, J. Spencer, C. J. Schofield, *J. Med. Chem.* **2013**, *56*, 6945.

[5] D. E. Dorman, L. J. Lorenz, J. L. Occolowitz, L. A. Spangle, M. W. Collins, F. N. Bashore, S. W. Baertschi, *J Pharm Sci* **1997**, *86*, 540.

[6] M. J. Frisch, G. W. Trucks, H. B. Schlegel, G. E. Scuseria, M. A. Robb, J. R. Cheeseman, G. Scalmani, V. Barone, B. Mennucci, G. A. Petersson, H. Nakatsuji, M. Caricato, X. Li, H. P. Hratchian, A. F. Izmaylov, J. Bloino, G. Zheng, J. L. Sonnenberg, M. Hada, M. Ehara, K. Toyota, R. Fukuda, J. Hasegawa, M. Ishida, T. Nakajima, Y. Honda, O. Kitao, H. Nakai, T. Vreven, J. A. Montgomery Jr., J. E. Peralta, F. Ogliaro, M. Bearpark, J. J. Heyd, E. Brothers, K. N. Kudin, V. N. Staroverov, R. Kobayashi, J. Normand, K. R. Raghavachari, A, J. C. I. Burant, SS Tomasi, J, M. Cossi, N. Rega, J. M. Millam, M. Klene, J. E. Knox, J. B. B. Cross, V, C. Adamo, J. G. Jaramillo, R Stratmann, RE Yazyev, O, A. J. Austin, R. Cammi, C. O. Pomelli, JW, R. L. M. Martin, K Zakrzewski, VG, G. A. Voth, P. Salvador, J. J. D. Dannenberg, S Daniels, AD Farkas, O Foresman, JB, J. V. Ortiz, J. Cioslowski, D. J. Fox, *Gaussian 09, revision D. 01*, Gaussian, Inc., Wallingford CT, **2009**.

[7] I. Funes-Ardoiz, R. S. Paton, *GoodVibes, v1.0.0*, DOI: 10.5281/zenodo.56091, **2016**.

[8] X. M. Zhang, F. G. Bordwell, M. Van Der Puy, H. E. Fried, *J. Org. Chem.* **1993**, *58*, 3060.

[9] Y. Zhao, D. G. Truhlar, *Theor. Chem. Acc.* **2008**, *120*, 215.

[10] J. Tomasi, B. Mennucci, R. Cammi, *Chem. Rev.* **2005**, *105*, 2999.

[11] C. P. Kelly, C. J. Cramer, D. G. Truhlar, *J. Phys. Chem. B* **2007**, *111*, 408.

[12] W. J. Li, D. F. Li, Y. L. Hu, X. E. Zhang, L. J. Bi, D. C. Wang, *Cell Res* **2013**, *23*, 728.

[13] J. D. Smith, M. Kumarasiri, W. Zhang, D. Hesek, M. Lee, M. Toth, S. Vakulenko, J. F. Fisher, S. Mobashery, Y. Chen, *Antimicrob Agents Chemother* **2013**, *57*, 3137.
